# Quality-controlled R-loop meta-analysis reveals the characteristics of R-loop consensus regions

**DOI:** 10.1093/nar/gkac537

**Published:** 2022-06-27

**Authors:** Henry E Miller, Daniel Montemayor, Jebriel Abdul, Anna Vines, Simon A Levy, Stella R Hartono, Kumar Sharma, Bess Frost, Frédéric Chédin, Alexander J R Bishop

**Affiliations:** Department of Cell Systems and Anatomy, UT Health San Antonio, San Antonio, TX, USA; Greehey Children's Cancer Research Institute, UT Health San Antonio, San Antonio, TX, USA; Bioinformatics Research Network, Atlanta, GA, USA; Department of Medicine, UT Health San Antonio, San Antonio, TX, USA; Center for Precision Medicine, UT Health San Antonio, San Antonio, TX, USA; Bioinformatics Research Network, Atlanta, GA, USA; Department of Biology, University of Ottawa, Ottawa, Canada; Bioinformatics Research Network, Atlanta, GA, USA; Faculty of Arts, University of Bristol, Bristol, U.K; Department of Cell Systems and Anatomy, UT Health San Antonio, San Antonio, TX, USA; Bioinformatics Research Network, Atlanta, GA, USA; Sam & Ann Barshop Institute for Longevity & Aging Studies, UT Health San Antonio, San Antonio, TX, USA; Glenn Biggs Institute for Alzheimer's and Neurodegenerative Diseases, UT Health San Antonio, San Antonio, TX, USA; Department of Molecular and Cellular Biology, UC Davis, Davis, CA, USA; Department of Medicine, UT Health San Antonio, San Antonio, TX, USA; Center for Precision Medicine, UT Health San Antonio, San Antonio, TX, USA; Department of Cell Systems and Anatomy, UT Health San Antonio, San Antonio, TX, USA; Sam & Ann Barshop Institute for Longevity & Aging Studies, UT Health San Antonio, San Antonio, TX, USA; Glenn Biggs Institute for Alzheimer's and Neurodegenerative Diseases, UT Health San Antonio, San Antonio, TX, USA; Department of Molecular and Cellular Biology, UC Davis, Davis, CA, USA; Department of Cell Systems and Anatomy, UT Health San Antonio, San Antonio, TX, USA; Greehey Children's Cancer Research Institute, UT Health San Antonio, San Antonio, TX, USA; May's Cancer Center, UT Health San Antonio, San Antonio, TX, USA

## Abstract

R-loops are three-stranded nucleic acid structures formed from the hybridization of RNA and DNA. While the pathological consequences of R-loops have been well-studied to date, the locations, classes, and dynamics of physiological R-loops remain poorly understood. R-loop mapping studies provide insight into R-loop dynamics, but their findings are challenging to generalize. This is due to the narrow biological scope of individual studies, the limitations of each mapping modality, and, in some cases, poor data quality. In this study, we reprocessed 810 R-loop mapping datasets from a wide array of biological conditions and mapping modalities. From this data resource, we developed an accurate R-loop data quality control method, and we reveal the extent of poor-quality data within previously published studies. We then identified a set of high-confidence R-loop mapping samples and used them to define consensus R-loop sites called ‘R-loop regions’ (RL regions). In the process, we identified a stark divergence between RL regions detected by S9.6 and dRNH-based mapping methods, particularly with respect to R-loop size, location, and colocalization with RNA binding factors. Taken together, this work provides a much-needed method to assess R-loop data quality and offers novel context regarding the differences between dRNH- and S9.6-based R-loop mapping approaches.

## INTRODUCTION

R-loops are three-stranded nucleic acid structures formed from the hybridization of RNA and DNA, often in regions with high G (guanine) or C (cytosine) skew, such as CpG islands (1–3). The study of R-loops has mostly focused on their pathological consequences in promoting genomic instability (4,5) and in hypertranscriptional cancers, such as Ewing sarcoma (6,7). However, recent studies have revealed that R-loops contribute to physiological processes, such as DNA repair (8), DNA methylation (9), and even reprogramming to pluripotency (10). While these studies have increased interest regarding the causes and consequences of physiological R-loops, fundamental questions regarding their subtypes, dynamics, and interactions remain unanswered.

R-loop mapping via high-throughput sequencing can elucidate R-loop locations and dynamics genome-wide. Presented in 2012, DNA:RNA immunoprecipitation (DRIP) sequencing was the first such technique, utilizing the S9.6 antibody to detect R-loops (2). In 2017, *Chen et al*. introduced ‘R-ChIP’, an R-loop mapping modality which relies on catalytically inactive RNase H1 (dRNH) as an alternative to S9.6-based methods (11). Though preliminary evidence suggested differences in dRNH and S9.6-based R-loop mapping (11,12), the extent and biological relevance of these disparities remains unclear. Recently, *Castillo-Guzman and Chedin* reviewed the relevant R-loop mapping literature and hypothesized that dRNH and S9.6-based modalities may identify biologically distinct subtypes of R-loops (‘Class I and II’) (13), but no studies to date have evaluated these subtypes bioinformatically. It is also unknown how different R-loop mapping modalities relate to each other more generally or whether they each reliably map the same sets of R-loops. This gap in knowledge has become a critical concern as, at the time of writing, there are now 23 distinct R-loop mapping modalities, 14 of which were developed since 2019 alone (Supplementary Table S1).

Previous work in the transcriptomics and epigenomics fields have demonstrated the potential for public data mining to yield novel biological insights (14,15). However, data mining studies are scant within the R-loop field. Our previous analysis of 108 public DRIP-Seq datasets (16) demonstrated the potential for greater insight from larger and more diverse datasets. However, it also revealed a number of inconsistencies in quality between publicly available R-loop mapping samples (16). These findings were echoed by the recent work of *Chedin et al*., who demonstrated that issues of data quality persist even within prominent R-loop mapping studies (17). While these findings indicate the need for robust quality control methods specific to R-loop datasets, no such techniques have yet been proposed to our knowledge.

In the present study, we profiled 810 publicly available R-loop mapping datasets. We devised methods to assess R-loop data quality control and applied these methods to identify high-quality R-loop mapping samples. From these samples, we defined consensus sites of R-loop formation, termed ‘R-loop regions’ (RL regions). From meta-analysis of RL regions, we observed stark contrasts between S9.6 and dRNH-based R-loop mapping results, supporting the concept of different subclasses of R-loops such as proposed with the ‘Class I/II’ hypothesis. We then explored R-loops mapped uniquely by dRNH, finding their enrichment at transcriptionally-silent, bivalent enhancer clusters in pluripotent stem cells. Finally, we characterized R-loop regions based on their level of conservation between independent samples, finding that they differ with respect to pathways relevant for transcription and replication. Moreover, we find that more constitutive RL regions are associated with housekeeping genes.

Taken together, our findings support a model in which dRNH-based mapping approaches are unable to measure most of the R-loop-covered genome, likely owing to the presence of RNA binding factors in these same regions. On the other hand, dRNH-based mapping approaches also have greater sensitivity to detect small, native R-loops in promoters and enhancers compared to S9.6 modalities. Though our model necessitates future wet-lab validation, it should prove useful for designing future R-loop mapping experiments.

In this study, we also establish novel approaches for the quality control and analysis of R-loop mapping data, which we provide in the collection of software tools, ‘RLSuite,’ which accompany this work (see Availability). While these software packages are not described in this work, we have made them publicly accessible, and we plan to provide a full description in a future manuscript.

## MATERIALS AND METHODS

### R-loop forming sequences (RLFS)

We predicted R-loop forming sequences using the *QmRLFS-finder* method (18) implemented as part of the *makeRLFSBeds.R* script in the accompanying data generation repository (see Availability). Briefly, we downloaded every genome for which gene annotations were available in ‘FASTA’ format from the University of California Santa Cruz (UCSC) genome repository. Then we ran the *QmRLFS-finder.py* script to generate predictions. Finally, we converted the results table to ‘BED’ format.

### Cataloguing R-loop mapping datasets

R-loop data was found by querying the Gene Expression Omnibus (GEO), Sequence Read Archive (SRA), ArrayExpress, and PubMed with the keywords ‘R-loop’, ‘R-loops’, ‘RNA:DNA hybrid’, ‘RNA:DNA hybrids’, ‘RNA-DNA hybrid’, ‘RNA-DNA hybrids’, ‘DRIP-Seq’, ‘R-ChIP’, ‘S9.6’ and ‘D210N’. We included all query results with R-loop mapping data publicly accessible via SRA. For every R-loop mapping sample, we recorded the following details:

1. The sample accession (GEO or SRA).
2. The accession for the corresponding genomic input sample (if available).
3. The sample condition (e.g., RNase H1 treated, ‘RNH’).
4. The mapping mode (e.g., ‘DRIP’ for DNA-RNA immunoprecipitation sequencing (DRIP-Seq)).
5. The tissue of the sample. For cell lines, this was the cell line name (e.g., ‘HEK293’). For organs and whole organisms, the tissue name (e.g., ‘B-cell’ for B-cells, ‘SC’ for whole brewer's yeast (Saccharomyces cerevisiae)).
6. The sample genotype, where relevant (e.g., ‘BRCA1’ for samples derived from BRCA1 mutant breast cancer patients).
7. ‘Other,’ a catchall for other relevant metadata provided by the original data submitters, such as drug treatment information.
8. The source publication PMID, if available.
9. The last author on the source publication, if available.

Supplementary Table S1 provides the full list of samples.

### Standardization of sample metadata

We standardized the manually curated catalogue via the custom scripts in the accompanying data generation repository (see Availability). First, the ‘condition’ was binarized to one of the following labels: ‘POS’ (samples which were expected to map R-loops, e.g., ‘D210N’ in R-ChIP data) or ‘NEG’ (samples which were not expected to map R-loops, for example, ‘Input’ for DRIP-Seq data). The mapping of ‘condition’ to ‘label’ relied on a manually curated regular expressions (REGEX) dictionary and simple string matching (Supplementary Table S1). Additionally, we filtered the manifest to keep only R-loop mapping samples (except for bisulfite sequencing samples). Finally, we executed the *RLPipes* v0.9.0 software program to query the SRA database for missing metadata related to each sample (see **Availability**).

### R-loop mapping data standardization

To reprocess all available R-loop mapping datasets, we used a long-running computational pipeline that is available in its entirety with detailed instructions in the accompanying data generation repository (see Availability). For all upstream processing steps, we used *RLPipes* v0.9.0, a CLI tool based on Snakemake (19) (see Availability). We executed this pipeline on a remote server running Ubuntu 18.04 with 500GB of RAM, 192 cores, and a 10GiB/s internet connection available. The pipeline took one week to finish processing all samples. The primary steps of the pipeline involved all the following:

First, raw reads in SRA format were downloaded for each SRA run via the prefetch software from NCBI *sra-tools*. Then, reads were converted to ‘FASTQ’ format using fastq-dump from sra-tools. Next, technical replicates were merged and interleaved (in the case of paired-end data) using *reformat.sh* from *bbtools* (20). Then, reads were trimmed and filtered with *fastp* (21), generating a quality report.

For R-loop mapping data, reads were aligned to the appropriate genome using bwa-mem2 (22). Then, alignments were filtered (minimum quality score of 10 enforced), sorted, and indexed using *samtools* (23) and duplicates were marked using *samblaster* (24). Then, peaks were called using *macs3* (25) and coverage was calculated using *deepTools* (26).

The outputs of the pipeline were (A) peaks in ‘broadPeak’ format, (B) coverage in ‘bigWig’ format, (C) quality reports for alignments and raw reads, and (D) alignments in ‘BAM’ format (see Availability).

### Normalization of read coverage

To calculate normalized coverage tracks for visualization purposes, we first calculated scaling factors using the trimmed mean of M-values (TMM) method previously demonstrated for similar sequencing approaches (27) implemented via the *edgeR* R package (28). Then, we calculated the expected fragment sizes for each sample using the *macs3 predictd* method (25). We then calculated coverage using the *bamCoverage* command from *deepTools* (26) with arguments ‘–scaleFactor <scale_factor> -e <fragment_size> –ignoreDuplicates’ where ‘<scale_factor>’ is the scaling factor derived from edgeR and ‘<fragment_size>’ is the predicted fragment size from *macs3*. The resulting tracks were visualized in the UCSC genome browser.

### R-loop mapping data quality control

Following data standardization, a quality control model was developed to filter out poor-quality samples prior to meta-analysis of R-loop consensus sites. This involved (1) R-loop forming sequences (RLFS) analysis, (2) quality model building and (3) sample classification.

#### R-loop forming sequences analysis

R-loop forming sequences (RLFS) analysis was performed using a custom R script, *rlfsAnalyze.R*, available in the accompanying data generation repository (see Availability). The analysis script calls the *analyzeRLFS* function from the *RLSeq* R package (see Availability). This function implements a method for assessing the enrichment of R-loop mapping peaks within RLFS via the following procedure: Permutation testing is implemented via the *permTest* function from the *regioneR* (29) R package such that, for each permutation, R-loop peaks were randomized using the *circularRandomizeRegions* function and then the number of overlaps with RLFS was counted. 100 permutations were used to build an empirical null distribution for peak/RLFS overlap. Then the true number of overlaps from non-randomized peaks and RLFS was compared to the null distribution to calculate the Z-score and significance of enrichment. Finally, a Z-score distribution was calculated using the *localZScore* function from *regioneR* (29) within 5kb upstream and downstream of the meta-RLFS midpoint. For more detail, see the *RLSeq* reference (see Availability). For visualization purposes, the scaled Z score distributions were calculated for each peak set in the reprocessed data and visualized using locally estimated scatterplot smoothing (LOESS) regression with the standard error as the confidence interval.

#### Quality model building

The quality classification model was built using the *models.R* script in the accompanying data generation repository (see Availability). The process of model building is semi-supervised and utilizes a graphical user interface to allow operators with minimal coding experience to conveniently use it. The model building procedure involves the following steps: (A) The operator executes the *models.R* script from the command line, launching an interactive web interface that contains a table with SRA experiment ID and label (‘POS’ or ‘NEG’) for each sample along with a plot showing the *Z*-score distribution from the RLFS analysis of that sample. This interface also indicates samples that have been previously deliberated about. (B) The operator then decides which samples to exclude from the model building process. A sample should only be excluded when the *Z*-score distribution drastically differs from the expected *Z*-score distribution for its label (examples are provided to the operator in the web interface). (C) Once all samples with a label mismatch are selected, the operator clicks the ‘Build model’ button which executes the model building process on the non-excluded data via the *RMarkdown* notebook, *FFT-classifier.Rmd* (available from the accompanying data generation repository). This notebook automatically executes the following steps: (C.1) The data are wrangled. (C.2) Then, the engineered features are calculated from the *Z*-score distributions (Table 1). The engineered features are then wrangled into matrix form with samples in rows and engineered features and label as the columns. The label in this case is ‘POS’ (expected to map R-loops) or ‘NEG’ (not expected to map R-loops). (C.3) Then, the feature matrix was standardized (centered and scaled) and transformed with the Yeo-Johnson normalization via the *caret* R package (30). The data was then partitioned using a 50:25:25 (train:test:discovery) split. (C.5) The discovery set was analysed with the Boruta automated feature selection method via the *Boruta* R package (31) to select the parsimonious feature set used to train the classifier. (C.6) The training set was then used to train a stacked ensemble classifier from the *caretEnsemble* R package (32), with the following architecture:

- The ensemble meta model is a Random Forest classifier and the five base models in the stack are:
- Latent Dirichlet allocation
- Recursive partitioning
- Generalized linear model (logit)
- K-nearest neighbours
- Support vector machine (radial)
- 10-fold cross-validation repeated 5 times was implemented during training.

**Table 1.** Engineered features used in the quality model. Where ‘*Z*’ is *Z*-score distribution, ‘ACF’ is the autocorrelation function, and ‘FT’ is the Fourier Transform (calculated via the *fft* function in R)

Feature	Description
***Z1***	mean of *Z*
***Z2***	variance of *Z*
***Zacf1***	mean of ACF(*Z*)
***Zacf2***	variance of ACF(*Z*)
***ReW1***	mean of FT(*Z*) (real part)
***ReW2***	variance of FT(*Z*) (real part)
***ImW1***	mean of FT(*Z*) (imaginary part)
***ImW2***	variance of FT(*Z*) (imaginary part)
***ReWacf1***	mean of FT(ACF(*Z*)) (real part)
***ReWacf2***	variance of FT(ACF(*Z*)) (real part)
***ImWacf1***	mean of FT(ACF(Z)) (imaginary part)
***ImWacf2***	variance of FT(ACF(*Z*)) (imaginary part)

(C.7) Finally, the model was evaluated on the test set with the *confusionMatrix* function from the *caret* R package (30).

#### Sample classification

Following model building, all samples were subsequently classified using the *classifySamples.R* script from the accompanying data generation repository (see **Availability**). This script primarily uses the *predictCondition* function from the *RLSeq* R package (see Availability). The procedure implements the following steps for each peak set in the reprocessed R-loop mapping data: (i) calculates the Fourier transform of the Z-score distribution, (ii) reduces the dimensions to the engineered feature set (see Quality model building), (iii) applies the pre-processing model to normalize these features (see Quality model building) and (iv) implements the classifier to render a preliminary quality prediction (see Quality model building). Of note, the quality model alone does not provide the final prediction for each sample. Instead, the final prediction is ‘POS’ (positive) only if all the following are true:

1. The RLFS Permutation test *P* value is significant (*P* < 0.05)
2. The *Z*-score distribution at 0 bp is greater than 0 (see R-loop forming sequences analysis)
3. The *Z*-score distribution at 0 bp is greater than the value at both −5000 bp (start) and +5000 bp (end) (see R-loop forming sequences analysis)
4. The quality model predicts a preliminary label of ‘POS.’

These conditions were chosen to ensure that a positive prediction from the model (based on the distribution of the *Z* score) also met baseline criteria which indicate enrichment in typical permutation testing (29). These criteria may prevent spurious predictions when the model is applied to data types on which it was not originally trained.

Finally, predictions were rendered for every sample for which peaks could be calculated (776/810 samples).

### QC model validation

To examine the internal and external validity of the predicted labels, several approaches were implemented: (A) RLFS consensus analysis, (B) genic feature enrichment analysis and (C) sample correlation analysis. All four (prediction:label) conditions: POS:POS, POS:NEG, NEG:POS, NEG:NEG were compared in each approach.

#### Visualization of normalized read coverage around MALAT1

This analysis involved four DRIP-Seq samples, ‘TCELL (Input)’ (SRX2455193), ‘TCELL’ (SRX2455189), ‘786-O (RNH)’ (ERX3974965), and ‘786-O’ (ERX3974964), in which both the ‘TCELL’ samples were from the same study (SRP095885), and the ‘786-O’ samples were also from the same study (ERP120322). Both ‘TCELL’ samples and both ‘786-O’ samples respectively were from the same study.

#### Feature enrichment analysis

Genic features were obtained via the *TxDb.Hsapiens.UCSC.hg38.knownGene* R package (33). The computationally predicted G4 quadruplexes (G4s) were provided by *Chariker et al*. in their recent work (34). The experimentally determined G4s were determined by *Chambers et al*. via their previous chromatin immunoprecipitation (ChIP) sequencing study (GEO accession: GSE63874) (35). All samples for which peaks could be called (34 samples excluded) were subjected to feature enrichment analysis for genic features and G4s. This analysis applied the following procedure to test the enrichment of peaks within these features: Using the *RLSeq* R package (i) peaks were randomly down sampled to a maximum of 10 000 peaks, (ii) down sampled peaks were overlapped with genic features, (iii) then, intersect statistics were calculated using Fisher's exact test via the *valr* R package (36), finally, (iv) log_2_ odds ratios of each label:prediction group were compared using a Kruskal–Wallis test and pairwise Dunn pos-hoc tests with Bonferroni p value correction, all performed via the *rstatix* R package (37).

#### Correlation analysis

This analysis uses correlation of R-loop signal around high-confidence R-loop sites to assess sample-sample similarity. To prepare the analysis, the read coverage track (bigWig) files for each sample were quantified around high-confidence sites (as first described by *Chedin et al*. (17)) via the *gsgCorr.R* script in the accompanying data generation repository (see Availability). First, high-confidence R-loop sites were identified from ultra-long-read R-loop sequencing (SMRF-Seq) (38). Then, high-confidence sites were lifted from hg19 to hg38 and extended by 100 kb bidirectionally. The sites were then binned into 1 kb windows. The read coverage signal for every human R-loop mapping sample was then summed over each bin to create a matrix of bins[i] x samples[j] containing the total signal of each sample within each bin [ij]. Finally, the *cor* function was used to calculate the Pearson correlation of the samples in the matrix. The results were visualized with the *ComplexHeatmap* R package (39).

### R-loop consensus analysis

This analysis identified human R-loop consensus sites within catalytically dead RNase H1 (dRNH) and S9.6-based samples. To identify general R-loop consensus regions (RL regions), the union of dRNH and S9.6 consensus sites was calculated. To obtain these data, the following procedure was implemented: First, samples were prepared for R-loop consensus analysis using the *prepareConsensus.R* script from the accompanying data generation repository (see Availability). The following criteria were used to select samples for R-loop consensus analysis: (i) label of ‘POS’, (ii) prediction of ‘POS’, (iii) at least 5000 peaks called under a *P* adjusted value of 0.05, (iv) is a human sample. We chose to enforce a label of ‘POS’ as ‘NEG’-labelled are subjected to treatments which should prevent robust R-loop mapping and may, therefore, introduce unwanted technical variance to the consensus analysis. We chose samples with at least 5000 peaks due to the need for random peak down sampling to 5000 ranges.

Two hundred and forty-seven samples met the full criteria for inclusion in the analysis of the 375 ‘POS’-labelled human samples. For each included sample, peaks were randomly shuffled and down sampled to 5000 ranges to ensure high peak-count samples would not dominate the analysis. Finally, samples were partitioned based on whether they used dRNH or S9.6-based R-loop mapping. Then, each down-sampled peak set was processed further using the *rlregions.smk* workflow (built on *snakemake*) from the accompanying data generation repository (see Availability).

For S9.6 and dRNH samples, the following were performed separately: (i) the peak sets were intersected with 10 bp genomic windows and the number of peak overlaps per window was counted (using *bedtools* (40)). (ii) The resulting windows and counts were converted to a bedGraph file (and its binary bigWig file format) and sorted using *bedtools* (40). (iii) *macs3 bdgpeakcall* (25) was then implemented with the options ‘-g 1000 -c x’ where $x\; = \;max( {floor( {.15*n\_samples} ),5} )\;$and n_samples is the number of samples provided. These steps yielded (A) consensus tracks (bigWig) and (B) consensus R-loop peaks (narrowPeak) for both S9.6 and dRNH samples.

Then, consensus RL Regions were generated using the *peakUnion.R* script from the accompanying data generation repository (see Availability). This script found the union of the peaks in the dRNH and S9.6 narrowPeak files, along with calculating the percent of samples within each group in which each peak appeared (conservation score) and averaging these scores in cases where dRNH and S9.6 peaks overlapped to create a peak union. Finally, RL regions and associated metadata were finalized using the *finalizeRLRegions.R* script from the accompanying data generation repository (see Availability). R-loops were filtered to remove any regions over 50kb in size, then every hg38 ‘POS’-labelled peak set was overlapped with the regions via the *bed_intersect* function from the *valr* R package (36) to yield a peak-RLRegion overlap matrix. This matrix was subsequently intersected with the sample metadata corresponding to each peak. Further details on additional metadata not used in this study are described in the *RLHub* R package reference (see Availability). Moreover, RL region ‘confidence scores’ were calculated for the purposes of colouring the RL region peaks when visualized in the genome browser. These scores are the geometric mean of scaled log_2_*pct_case* (percent of samples detecting the RL region which are predicted ‘POS’), *nStudies* (number of independent studies in which the RL region is detected), log_2_*medQVal* (the median false discovery rate of peaks which overlap this RL region), and the log_2_*medSignalVal* (the median signal value of peaks which overlap this RL region).

### Consensus site signal around genes

Consensus R-loop signal for dRNH and S9.6 was quantified around all hg38 ensemble genes using the *computeMatrix* function from the *deepTools* software program (26). The resulting data was scaled based on the number of samples in each group. The scaled signal was then scaled to a [0, 1] range prior to plotting.

### Consensus overlap analysis with genomic annotations

The following analyses were performed automatically by running the *figures.R* script which accompanies this work (see Availability).

#### Peak summitting

For analyses which involved calculating the overlap (with or without enrichment analysis) of dRNH and S9.6 consensus peaks with genomic features, peaks were ‘summited’ (length-standardized) prior to overlap to mitigate size biases. Standardization involved finding the position of highest signal within the peaks (peak summit) and extending 250 bp bidirectionally to form 500 bp ranges. Peak summits were provided automatically by the output of *macs3* peak calling. Summited peaks were then overlapped with genomic annotations for downstream analysis via the *bed_intersect* function from the *valr* R package.

#### Transcript feature overlap

Transcript features (tx features) were obtained from the *TxDb.Hsapiens.UCSC.hg38.knownGene* R package (33). Peak overlap with tx features was calculated via the *bed_intersect* function from the *valr* R package. Intersected peaks and tx features were analysed and a unique transcript feature was assigned to each peak. To achieve unique assignment, a priority rule was enforced such that peaks which overlapped two tx feature types would be assigned to the one with the highest priority. The priority order was ‘TSS’, ‘TTS’, ‘fiveUTR’, ‘threeUTR’, ‘Exon’ and ‘Intron’. The ‘Intergenic’ assignment was provided to all peaks which did not intersect any tx feature.

### Enhancer analysis

#### Annotation sources

Enhancer annotations were developed from three sources: predicted cis-regulatory elements (CREs) provided by ENCODE SCREEN (41), high-confidence enhancers and enhancer-gene interaction data provided by GeneHancer v4.4 (42), and induced pluripotent stem cell (iPSC) chromatin states (ChromHMM (43) 18-state models) provided by ENCODE (file set: ENCSR976ZHN) (41). Statistical enrichment of peaks within CREs was calculated using the *bed_fisher* function from the *valr* R package (36). Enrichment metaplots within GeneHancer enhancers was calculated via the *binOverFeature* function from the *ChIPpeakAnno* R/Bioconductor package (44). Distal enhancers were identified using the *annotatePeak* function from the *ChIPseeker* R/Bioconductor package (45).

#### Cell type-specific analysis of enhancer regions

To analyze the relationship between enhancers and R-loops mapped by dRNH modalities, cell type-specific global run-on (GRO) sequencing datasets were obtained from the Gene Expression Omnibus (GEO) in induced pluripotent stem cell (iPSC) (GSE117086) and CUTTL1 (GSE115894) cell lines. These were analysed jointly with the MapR (a dRNH-based R-loop mapping modality) datasets curated previously via the steps described beforehand (see R-loop mapping data standardization). Samples were selected which matched the same confidence filters applied in the generation of consensus peaks (see R-loop consensus analysis). Gene set over-representation analysis was implemented to identify the pathways relevant to R-loop bound distal enhancers in each cell line. Briefly, the distal enhancers overlapping intergenic MapR peaks were identified. Then, the GeneHancer interaction database was queried to obtain high-confidence gene targets for each enhancer. Gene set over-representation proceeded using the *enrichr* function from the *enrichR* R package (46,47) with gene sets from the CellMarker database (48), the MSigDB Hallmark database (49), the ChEA database (50) and the ARCHS4 transcription factor database (51).

#### Analysis of CTCF/Cohesin co-localization with R-loops

To analyze the relationship between CTCF/Cohesin and R-loops, cell type-specific CTCF ChIP-sequencing datasets were obtained from the ENCODE in iPSC (ENCFF322WKG) cells and from GEO for CUTTL1 (GSE130140) cells. We also obtained HiC sequencing data from GEO for iPSC cells (GSE125540) (CUTLL1 not available). HiC-sequencing data was added to the UCSC genome browser and compared with MapR bigWig tracks. Enrichment analysis of CTCF peaks within MapR peaks was calculated via the *binOverFeature* function from the *ChIPpeakAnno* R/Bioconductor package. CTCF, RAD21 and SMC3 consensus peaks were obtained from the ENCODE3 transcription factor binding site collection, provided by the UCSC table browser. SA1 and SA2 peaks were curated as previously described (16), and then consensus peaks were calculated using the *chip-r* command line utility (52). R-loop consensus peaks were enriched within CTCF peaks using the *binOverFeature* function. Then, Fisher's exact tests were performed using the *bed_fisher* function from the *valr* R package for R-loops within CTCF, RAD21, SMC3, SA1, and SA2 peaks.

#### Bivalent enhancer analysis

GeneHancer enhancer annotations were extended to include the labels from the chromHMM 18-state model predictions in iPSC cells (described in preceding section). Briefly, chromHMM enhancer states were overlapped with distal GeneHancer enhancers using the *findOverlapsOfPeaks* function from the *ChIPpeakAnno* R/Bioconductor package (44). Overlapping chromHMM enhancer labels were transferred to the GeneHancer enhancer annotations for further analysis. Global Run-on (GRO) sequencing, RNA Pol II ChIP sequencing, CTCF ChIP sequencing, EZH2 ChIP sequencing, ATAC sequencing, H3K27me3 ChIP sequencing, H3K4me1 ChIP sequencing, H3K27ac ChIP sequencing, and HiC sequencing datasets were obtained from GEO for the hg19 human genome and input into a custom genome browser session (for a list of accessions, see Availability). Tornado plots were constructed using the *computeMatrix* and *plotHeatmap* functions from the *deepTools* package on chromHMM-predicted bivalent (or non-bivalent) enhancers (26).

#### ChIP/eCLiP enrichment analysis

To analyze the location of S9.6 and dRNH consensus peaks compared to sites of chromatin and RNA localization of RNA binding proteins, data was downloaded from the ENCODE database using the accessions recently described (53). Enrichment of peaks within ChIP/eCLiP sites was calculated using the *bed_fisher* function from the *valr* R package (36).

### R-loop region abundance calculation

R-loop region (RL region) abundance was calculated within each human sample using the *rlregionCountMat.R* script from the accompanying data generation repository (see Availability). sample alignment files (‘BAM’ format) were processed with *featureCounts* from the *Rsubread* R package to quantify the read counts from each within RL regions (54). Finally, the variance stabilizing transform (VST) was used to calculate normalized counts (55).

### Differential abundance of RL regions

Differential abundance between dRNH and S9.6 samples was calculated by the following procedure: (1) the count matrix was subset to contain only the RL regions found by both dRNH and S9.6 peaks, (2) the *DESeq* function from the *DESeq2* R package (55) was applied to calculate differential abundance, (3) the *enrichR* R package (46) (and R interface to the *enrichr* web service (47)) was used to find the enrichment of gene ontology (GO) (56) terms within the genes overlapping differentially abundant RL regions.

Additionally, the *prcomp* function in R was used to calculate principal component analysis plots and the *EnhancedVolcano* R package (57) was used to generate the volcano plot. Downsampled permutation testing was performed by randomly downsampling the S9.6 dataset to include the same number of studies as the dRNH dataset. Then, if the S9.6 dataset still contained more samples than dRNH, it was downsampled again to include the same number of samples. This approach was designed to ensure that dRNH and S9.6 downsampled datasets contained both the same number of studies and (approximately) the same number of samples. Random permutations were performed 10 times, and the differential R-loop abundance was recalculated with each. Finally, the Pearson correlation of the differential abundance Wald statistic was compared pairwise between all samples and visualized with the *pheatmap* R package.

Finally, the pausing index for each gene (58) was calculated using the *getPauseIndices* function from the *BRGenomics* package (59). This calculation was performed using precision run-on (PRO) sequencing data in multiple cell lines, downloaded from different sources (see Accessions).

### Conservation analysis of RL regions

Conservation (percent of samples which detect an RL region) was ranked and bins were selected to partition RL regions by conservation percentiles. Binned RL regions were overlapped with genomic features and enrichment was calculated via the procedure described above (see Consensus site overlap in genes). Pathway enrichment was also performed via the procedure outline in the above section (Differential abundance of RL regions), with the only difference being that the ChEA (50), KEGG (60), and MSigDB (49) pathway databases were tested.

## RESULTS

### Curation of public R-loop mapping samples

The data in this study were hand-curated from public data repositories, yielding 810 R-loop mapping samples from 74 different studies (Supplementary Table S1). The data are technologically and biologically diverse, representing 67 different tissue types, seven species, and 20 R-loop mapping modalities (Supplementary Table S1, Figure S1A). Moreover, the data includes both ‘POS’ (expected to map R-loops) and ‘NEG’ (not expected to map R-loops) labelled samples (Supplementary Table S1, Figure S1A). Examples of NEG-labelled samples include ‘WKKD’ (RNase H1 that is incapable of both DNA binding and R-loop resolution (11)) R-ChIP data, RNase H1-treated DRIP-Seq, and genomic ‘Input’ samples. Examples of POS-labelled samples include ‘D210N’ (catalytically inactive RNase H1) R-ChIP data and typical S9.6 DRIP-Seq data. The scale and diversity of these data made them suitable as a basis for building our quality methods and for our subsequent meta-analyses.

**Figure 1.**
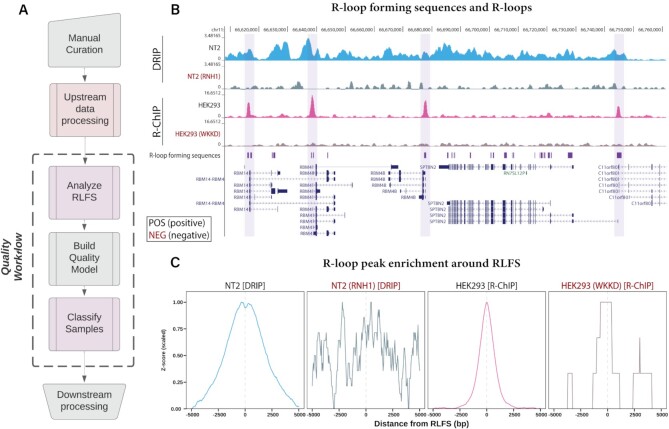
Reprocessing and standardization of R-loop mapping data reveals enrichment within R-loop forming sequences (RLFS). (**A**) The workflow used for reprocessed datasets. (**B**) A Genome Browser view of four representative samples. One ‘POS’-labelled (expected to map R-loops) and one ‘NEG’-labelled (not expected to map R-loops) sample each from DRIP and R-ChIP modes along with R-loop forming sequences (RLFS). NEG-labelled samples are indicated with red text (e.g. ‘RNH1’). The genome browser session for this visualization is also provided within this manuscript (see Availability). The SRA IDs of these samples are SRX1025894 (‘NT2’), SRX1025896 (‘NT2 (RNH1)’), SRX2683605 (‘HEK293’), SRX2675009 (‘HEK293 (WKKD)’; WKKD: mutant RNase H1 without catalytic or DNA binding capabilities). (**C**) Metaplots of the four samples in (B) showing the enrichment of their called peaks around RLFS. The Y axis is the *Z* score after min-max scaling.

Following manual curation of R-loop mapping datasets, we developed a purpose-built R-loop pipeline tool called ‘RLPipes’ and applied it to reprocess all datasets, yielding peaks, read coverage and other processed files (see Availability) (Figure 1A).

### The number of R-loops detected varies within and between modalities

Previous work has demonstrated discrepancies in the number of R-loop sites uncovered by different mapping methods (12). However, to our knowledge, no study to-date has quantified the extent of these differences. From our analysis of the numbers of peaks called from various techniques in POS-labelled samples, we found discrepancies between modalities spanning multiple orders of magnitude (Supplementary Figure S1B). We found that DNA:RNA in vitro enrichment (DRIVE) sequencing produced the fewest peaks (337). However, only one DRIVE-Seq sample is publicly available at present (1). Following DRIVE-Seq, R-ChIP contains the fewest average peaks (∼6 thousand). Strand-specific DRIP (ssDRIP) sequencing generated the greatest average number of peaks (∼314 thousand). Moreover, we observed variability among the number of peaks even within mapping modalities (Supplementary Figure S1B). Taken together, these findings suggest that no single modality can confidently assess the locations of all R-loops within a sample. The results also reveal technical variance between samples belonging to the same mapping modality, suggesting potential issues with sample quality control across studies.

### R-loop forming sequences provide a suitable test of sample quality

Recent studies have identified the presence of poor-quality R-loop mapping datasets within the literature (16,17), but no tests of R-loop mapping accuracy have yet been proposed to our knowledge. To address this limitation, we developed a purpose-built quality control approach based on R-loop forming sequences (RLFS). RLFS are genomic regions which show favourability for R-loop formation (18,61–64). They are computationally predicted from genomic sequence with tools like QmRLFS-finder (18). We calculated RLFS across each genome and found that they agreed well with the R-loop mapping data we reprocessed (Figure 1B). We then implemented permutation testing to assess the statistical enrichment of R-loop peaks within RLFS. From this analysis, we found a strong enrichment within RLFS for representative POS-labelled samples and a lack of enrichment within NEG-labelled samples (Figure 1C). These findings highlighted the suitability of using RLFS as a basis for assessing R-loop mapping data quality.

### Ensemble learning from RLFS provides accurate quality predictions

Evidence from our recent work and from *Chedin et al*. indicated that POS-labelled samples may sometimes fail to map R-loops as expected (16,17). To address this concern, we performed RLFS analysis for every sample with peaks available (776 samples), yielding a *P* value and *Z* score distribution for each. As anticipated, we found some samples with a ‘POS’ label, but which did not show expected enrichment around RLFS, based on *Z* score (Supplementary Figure S2A). This indicated the potential for using RLFS analysis to identify POS-labelled samples which fail to map R-loops as expected.

We reasoned that the p value from RLFS analysis would be sufficient for distinguishing sample quality. Unexpectedly, we found that while many POS-labelled samples showed the expected *Z*-score distribution and significant *P* value, there were also examples of false positives in which the *P* value was significant, but poorly representative of both the assigned label and of the *Z* score distribution (Supplementary Figure S2B). This indicated the need for a more sophisticated test of sample quality from the RLFS analysis results, prompting our development of an ensemble learning model which could analyze the ‘noisiness’ of the *Z* score distribution to provide additional discriminatory capability.

We built a classifier using a semi-supervised approach that involves a human operator deciding whether to exclude samples from the training set based on the alignment between the RLFS Z-score plot and the sample label (Supplementary Figure S2C, D). The purpose of this supervision was to prevent the model from learning the ‘POS’ label from samples which likely failed to map R-loops and *vice versa* with the ‘NEG’ label. Of the 776 samples which had sufficient read depth for peak calling, the operator excluded 157 (20.2%) due to a mismatch between the sample label and the *Z* score distribution. Having selected the samples to discard, the operator triggered the automated model building script, which initiated the training process.

Most features in the discovery set were automatically selected using the Boruta method (31) (Supplementary Figure S2E). The ensemble learning model was trained via the automated script and evaluated internally via repeated cross validation, showing average performance above 0.95 specificity, 0.85 sensitivity, and 0.95 receiver operator characteristics area under the curve (ROC) for all base models (Supplementary Figure S2F). The final model performance was evaluated on a test set which the model had never previously seen. On this data, the model displayed an accuracy of 0.9191 (95% confidence interval: 0.8599-0.95189; no information rate (NIR): 0.625; *P*-value [accuracy > NIR]: 3.58e–15) (Supplementary Figure S2G), demonstrating the high accuracy of the model.

Finally, we combined the machine learning model with three other assessments to develop our final quality model. Briefly, a sample with a final prediction of ‘POS’ must meet all the following criteria: (i) have a permutation testing *P* value below 0.05, (ii) show a *Z* score above 0 at 0 bp in the distribution, (iii) show a *Z* score at 0 bp which is greater than both the *Z* score at –5 kb and +5 kb, and (iv) receive a prediction of ‘POS’ from the machine learning model. In this work, ‘quality model’ refers to criteria i–iv. Of note, the purpose of criteria i–iii is to ensure that a ‘POS’ prediction from the machine learning model also meets baseline standards for assessing permutation testing results (29).

### Quality model is internally valid and highlights ‘false negative’ samples

**Figure 2.**
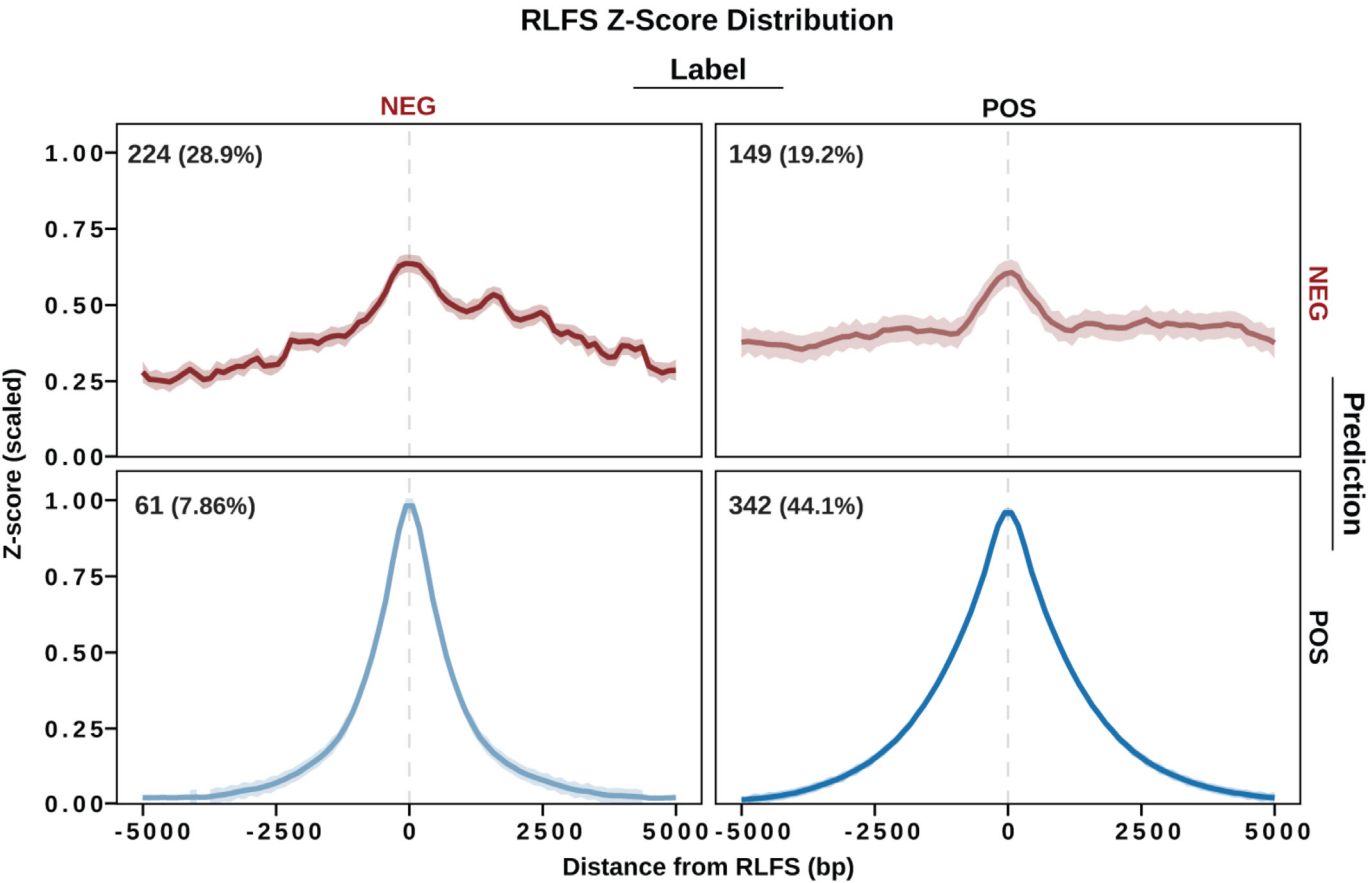
QC model predicts incorrectly labelled (false negative/positive) R-loop mapping datasets based on enrichment within R-loop forming sequences. Metaplots display the consensus signal among samples that were labelled ‘POS’ (expected to map R-loops) or ‘NEG’ (not expected to map R-loops) and samples which were predicted by the quality model to be ‘POS’ or ‘NEG’. The plots show the LOESS regression line along with a confidence interval based on the standard error from LOESS regression. The annotations show the number and percentage of samples which are included.

We then applied the quality model to classify every reprocessed dataset in the present study (Figure 2). Unexpectedly, we found a discrepancy between the assigned label (based on author-supplied metadata) and the predicted label (based on our quality model) for many samples (Figure 2). Of the 285 NEG-labelled samples, the quality model classified 61 (21.4%) as ‘POS’ (‘false negatives’). Of the 491 POS-labelled samples, the quality model classified 149 (30.35%) as ‘NEG’ (‘false positives’). Moreover, we noticed that some modalities were more likely than others to contain these ‘false negatives’ and ‘false positives’ (Supplementary Figure S2H). To better understand these results, we divided the data by all prediction:label combinations (NEG:NEG, NEG:POS, POS:NEG, POS:POS) and calculated the consensus *Z* score distribution for each group. The results demonstrate that the classifications robustly represent the RLFS analysis of the data (Figure 2, Supplementary Figure S3A). Coupled with the high accuracy of the ensemble model (Supplementary Figure S2G), these findings demonstrate that our quality model is *internally* valid.

Of note, we observed that the model classified 61 ‘NEG’-labelled samples as ‘POS’ (‘False negatives’). From univariate analysis, we found that most false negatives (50/61; 82.0%) occur in samples treated with RNase (H1, A, or T) or actinomycin D, suggesting incomplete R-loop degradation (Supplementary Figure S3B). Surprisingly, among the remaining 11 we noticed two DRIP-Seq input controls (Supplementary Figure S3B). DRIP-sequencing input controls model the genomic background and capture biases in restriction enzyme digestion. Therefore, we did not expect to find input samples enriched within RLFS. To address this finding, we first confirmed that these two samples were both enriched within RLFS (Supplementary Figure S3C), and then proceeded to examine them on the genome browser alongside example ‘true positive’ (POS:POS) and ‘true negative’ (NEG:NEG) samples (Supplementary Figure S3C). Curiously, we found that the signal from these samples was not randomly distributed, as we would have expected from a typical genomic input control. Moreover, we observed that input control signal summits coincided with promoter regions and CpG islands (Supplementary Figure S3C), suggesting a bias that may be related to sequence content. While these false negatives represent a slim minority of the dataset, they highlight the importance of background controls when designing R-loop mapping experiments so that bioinformaticians can reduce bias during peak calling.

### Validation of quality model reveals the prevalence of poor-quality R-loop mapping data

**Figure 3.**
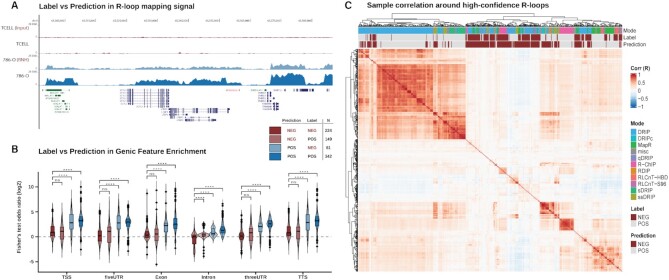
Quality control model predictions are externally valid. (**A**) A Genome Browser image capture showing examples of DRIP-Sequencing datasets from two studies that exemplify all four possible quality model prediction and sample label (prediction:label) combinations (‘TCELL (Input)’ [SRX2455193] - NEG:NEG; ‘TCELL’ [SRX2455189] - NEG:POS; ‘786-O (RNH)’ [ERX3974965] - POS:NEG; ‘786-O’ [ERX3974964] - POS:POS). The genome browser session for this visualization is also provided within this manuscript (see Availability). (**B**) Violin/Box plots showing the distribution of Fisher's exact test odds ratios within all reprocessed R-loop mapping samples, split by prediction:label combination and by the genomic feature on which testing was performed. Significance was determined via the Kruskal-Wallis test followed by Dunn post-hoc with Bonferroni correction. ****P < 0.0001; ***P < 0.001; **P < 0.01; *P < 0.05; ns = *p* ≥ 0.05. (**C**) A heatmap showing the Pearson correlation (*R*) of reprocessed R-loop mapping samples around high-confidence R-loop sites. Dendrograms and row order show hierarchical clustering of samples and annotations show the prediction, label, and mode of each sample.

We next proceeded to assess the *external* validity of our quality method through other quality approaches that did not involve RLFS analysis. The first approach was to visualize the normalized read coverage tracks at sites of known R-loop formation. A representative depiction of four DRIP-Seq samples at a region near *MALAT1* (a non-coding RNA with constitutive R-loop formation (65)) shows that the model predictions distinguish between samples which display R-loop detection and those which do not (Figure 3A). These results provided preliminary evidence for the external validity of our approach for determining whether a sample maps R-loops as expected. Of note, this analysis also highlights an example of incomplete RNase H1 treatment in DRIP sequencing data, ‘786-O (RNH)’ (Figure 3A), as discussed in the previous section.

We then implemented enrichment testing for genic features, such as exons and introns, across all human samples in each prediction:label group. Given that R-loops are a by-product of normal transcription, successful R-loop mapping should result in an enrichment of peaks within genic features, as has been shown previously (11,16,66). From our analysis, we found that NEG-predicted samples, regardless of their original label, typically had low enrichment in all genic features (Figure 3B). Moreover, we observed that the enrichment of genic features (with the exception of ‘Intron’) was not significantly greater in NEG:POS (prediction:label) samples compared to NEG:NEG samples (Figure 3B). Conversely, POS:NEG showed a significant increase in enrichment in all features when compared to NEG:NEG (Figure 3B). Together, these results support the external validity of our model predictions.

We then repeated the same analysis with both predicted and experimentally determined G4 quadruplexes (G4s) (Supplementary Figure S4A). G4s are non-B DNA structures which may promote R-loop stability when present on the displaced ssDNA strand (67–69). From feature enrichment analysis of G4s, we found that POS-predicted samples were significantly more enriched than NEG-predicted samples, regardless of prior label (Supplementary Figure S4A).

Previous work by Ginno *et al* demonstrated that G/C skew (i.e. G or C skew) is a common feature of unmethylated CpG islands (CGIs) with R-loop occupancy (2). Thus, we would expect to observe an enrichment of successfully mapped R-loops within both CGIs and regions of G/C skew. We obtained predicted CGI locations from the UCSC table browser, and we predicted G/C skew regions using the *SkewR* algorithm on medium-confidence mode (2). As expected, we observed a significant enrichment of POS-predicted samples within CGIs and G/C skew regions when compared to NEG-predicted samples, regardless of prior label (Supplementary Figure S4B). Taken together, these findings further demonstrate the external validity of the model predictions and their applicability to the analysis of R-loop mapping results in a biological context.

Finally, we implemented sample-sample correlation analysis to evaluate the agreement between the results obtained from the model prediction and the sample-level similarity as determined through correlation (Figure 3C). As expected, we found that NEG-predicted samples tended to cluster together and display low correlation or no correlation with most POS-predicted samples (Figure 3C). Likewise, we found that POS-predicted samples tended to cluster together, regardless of their original metadata label (Figure 3C). While these trends were broadly consistent, there were notable exceptions in which NEG-predicted samples correlated with POS-predicted samples, thus representing disagreement between the correlation analysis and the quality model predictions (Figure 3C). However, we expected to observe a small amount of disagreement, as the correlation analysis relies upon a fundamentally different approach from the quality model. Moreover, the finding that these approaches agree in most cases lends further support to the argument that the quality model is externally valid. Finally, these results imply that a comprehensive evaluation of R-loop data quality may benefit from the implementation of overlapping and complementary quality control methodologies.

Taken together, these internal and external validations demonstrate that the method we present here is an accurate measure of R-loop mapping data quality. They also reveal the extent of poor R-loop data within published datasets, indicating that the approach described here will be of great benefit to future studies. Of note, we provide access to this quality method in the *RLSeq* R package (see Availability).

### Consensus analysis reveals the locations and prevalence of R-loop formation

The locations and prevalence of R-loops genome-wide has been a subject of study since the first R-loop mapping modalities were described (1,66). However, current estimates of where R-loops form and the percentage of the genome which they cover (3–13%) are derived from the analysis of small numbers of samples and mapping technologies (66,70,71). As we demonstrate here, there is variation in the number of peaks called both between and within modalities (Supplementary Figure S1B), indicating that the true prevalence and locations of R-loops remain unclear. Moreover, R-loop mapping experiments which rely upon the S9.6 antibody identify different R-loops from those which rely upon catalytically dead RNase H1 (dRNH) (11,12). This has caused some to raise the intriguing possibility that S9.6 and dRNH-based mapping experiments might detect different ‘classes’ of R-loops (13), though the characteristics of these R-loop classes remains poorly understood.

**Figure 4.**
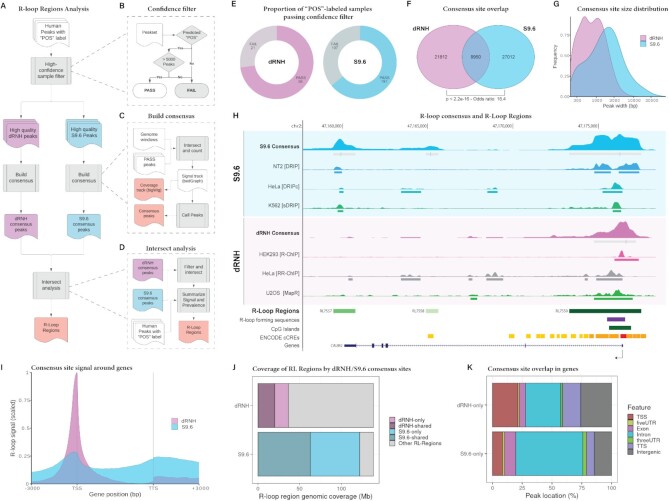
Consensus analysis of high-quality samples reveals differences in dRNH and S9.6-mapped R-loops. (**A**) A flow diagram showing the workflow used for consensus analysis of R-loop sites in catalytically dead RNase H1 (dRNH) mapping samples and S9.6-based (S9.6) mapping samples. (**B**) A flow diagram showing the algorithm for selecting high-confidence samples to include in the analysis. (**C**) A flow diagram showing the workflow for building consensus R-loop signal and calling consensus peaks. (**D**) A flow diagram showing the workflow for intersect analysis, which yields R-Loop Regions. (**E**) Donut charts showing the proportion of ‘POS’-labelled dRNH and S9.6 samples passing the quality filters. (**F**) A Venn diagram showing the overlap of consensus sites derived from dRNH and S9.6-based mapping samples. P value and odds ratio from Fisher's exact test. (**G**) Density plot showing the frequency of consensus peak width derived from S9.6 and dRNH-based mapping samples. X axis is log_10_ scaled. (**H**) Genome browser view of a top R-loop region (at the *CALM2* gene) with dRNH and S9.6 consensus signal, peaks, and signal tracks/peaks from representative S9.6 and dRNH R-loop mapping samples. ‘R-loop regions’ were derived from union of dRNH/S9.6 consensus peaks and are coloured based on the ‘confidence score’ of that consensus (see Materials and Methods). Arrow indicates direction of transcription. ENCODE cCREs are tissue agnostic cis regulatory elements predicted by ENCODE-SCREEN, provided by the UCSC genome browser (colour code: red is ‘promoter-like signature’; orange is ‘proximal enhancer-like signature’; yellow is ‘distal enhancer-like signature’). The genome browser session for this visualization is also provided within this manuscript (see Availability). (I) Metaplot showing the R-loop consensus signal (scaled by total number of samples in dRNH and S9.6 respectively) around genes. (**J**) Stacked bar chart showing the proportion of total RL Region genomic coverage (in megabases, ‘Mb’) occupied by S9.6 and dRNH sites. S9.6 and dRNH consensus sites are split into ‘only’ (unique to either S9.6 or dRNH) and ‘shared’ (detected by both S9.6 and dRNH). (K) Annotation plot showing the proportion of summitted (500 bp width) dRNH and S9.6 consensus sites within various genomic features.

To address these gaps in knowledge, we used high-confidence human R-loop mapping samples to generate dRNH- and S9.6-specific R-loop ‘consensus regions’ (Figure 4A-D). We first defined a high confidence set of samples based on the label, prediction, and number of peaks in the sample (Figure 4B). We found that 56 of 77 human POS-labelled dRNH samples and 191 of 298 human POS-labelled S9.6 samples passed the quality filters (Figure 4E). The analysis proceeded as described (see Methods) and yielded consensus sites and signal for dRNH and S9.6 separately, along with a consensus set of ‘R-loop Regions’ (RL regions) from the union of S9.6 and dRNH consensus sites (see Availability). Of note, each valid ‘consensus site’ was observed independently in at least 15% of all samples for both dRNH (8 out of 56 required) and S9.6 (28 out of 191 required). This constraint increases confidence that the consensus sites represent genuine R-loop formation.

A recent study from Lin *et al* employed a consensus analysis approach to identify high-confidence ‘R-loop zones’ (72). However, this approach does not consider dRNH and S9.6 samples independently, and it does not use an R-loop-specific QC model to ensure sample quality prior to analysis (72), making it unsuitable for the present study.

From analysis of R-loop consensus regions, we observed that dRNH samples yielded 34,451 consensus sites while S9.6 samples yielded 37,043 consensus sites. These sites collectively cover 138.6 megabases (Mb), which is 4.32% of the human genome (dRNH: 36.4Mb, 1.13% of genome; S9.6: 121.8Mb, 3.80% of genome). Moreover, we calculated the overlap of these sites and found 9950 shared sites (63.3Mb, 1.97% of genome) (Figure 4F). This indicated that despite having clear differences, dRNH identified many of the same R-loops as S9.6. Moreover, we observed that S9.6 consensus peaks tended to be larger than dRNH consensus peaks (Figure 4G).

We then examined consensus peak signal at *CALM2*, a gene containing one of the most mapped R-loops in our analysis. Interestingly, we observed that while both S9.6 and dRNH peaks localize around the transcription start site (TSS), only signal from S9.6 was found in the gene body and around the transcription termination site (TTS) and Poly-A regions (Figure 4H). This finding led us to perform a meta-analysis of dRNH and S9.6 consensus sites within genic features (Figure 4I–K). First, we identified the consensus peaks found uniquely by S9.6 and dRNH, noticing that ∼75% of S9.6 peaks do not co-localize with any dRNH peaks (‘S9.6-only peaks’) and roughly 65% of dRNH peaks do not co-localize with any S9.6 peaks (‘dRNH-only peaks’) (not shown). From analysis of the 4.32% of the genome covered by RL regions, S9.6 consensus peaks occupy most of those regions (87.9% of total RL region genomic coverage) (Figure 4J). Conversely, we found that dRNH consensus peaks only cover 26.3% of RL Regions (Figure 4J). Moreover, comparing where S9.6- and dRNH consensus sites overlap (‘dRNH/S9.6-shared’ sites), we find that S9.6 consensus sites identify a far larger area of genomic coverage (Figure 4J).

These results suggested a disparity regarding the proportion of the R-loop-occupied genome which is accessible by dRNH compared with S9.6. One potential explanation might be that the efficiency of R-loop mapping within certain genomic features is higher for S9.6 compared to dRNH and *vice versa*. From analysing the dRNH-only and S9.6-only peaks with respect to genic features, we observed that while both S9.6 and dRNH show a preference for the TSS and TTS of genes, dRNH is more specific to these regions than S9.6 (Figure 4K). Likewise, we observed that S9.6-only regions were more specific to intronic sequences (Figure 4K).

Taken together, these findings indicated that consensus R-loop sites recapitulate expected R-loop biology. Moreover, they reveal strong differences between dRNH and S9.6 mapping approaches with respect to the proportion of the genome and the genomic features in which they detect R-loops.

### Differential analysis defines Class I/II R-loops based on association with promoter pausing

In their recent work, *Castillo-Guzman and Chedin* hypothesized the existence of two previously unstudied classes of R-loops: ‘Class I’ (R-loops resulting from *promoter proximal pausing*) and ‘Class II’ (R-loops resulting from *transcriptional elongation*) (13). They hypothesized that Class I R-loops are mapped by dRNH-based experiments with high efficiency, but that these experiments are unable to efficiently map Class II R-loops (13). In the above analysis of S9.6 and dRNH consensus sites, we found support for this hypothesis by showing that dRNH consensus signal aggregates around the TSS region while S9.6 signal covers the gene body (Figure 4I, K). Moreover, our finding that dRNH-mapped R-loops occupy a much smaller proportion of the genome than S9.6-mapped R-loops also fits with this hypothesis (Figure 4J). However, we had not yet addressed a central tenet of the Class I/II hypothesis—namely, that dRNH binds R-loops which result from promoter proximal pausing. Moreover, we had not yet addressed the genes in which Class I/II R-loops form, nor the biological programs in which those genes participate.

**Figure 5.**
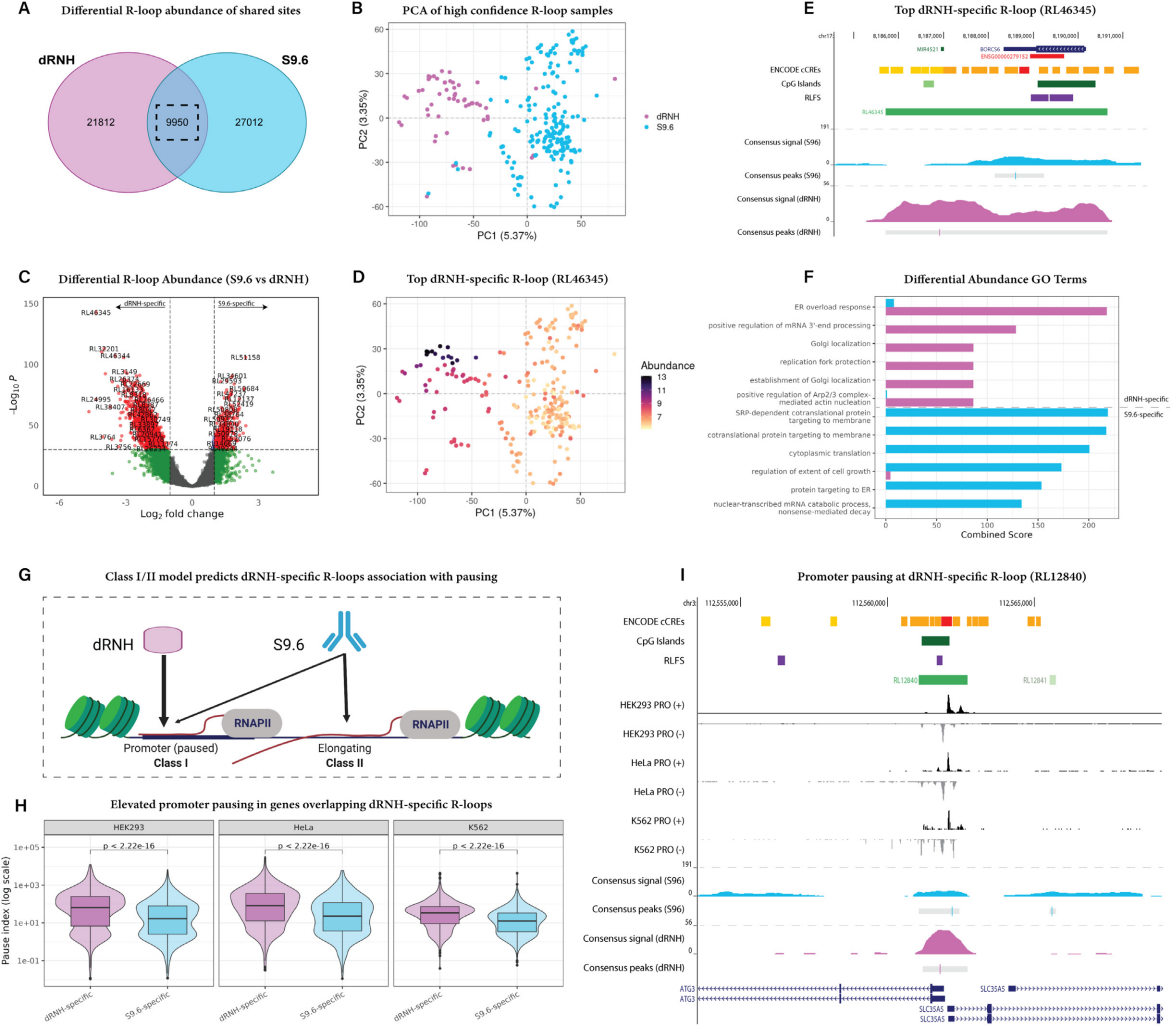
Differential abundance analysis of dRNH- and S9.6-detectable R-loop regions reveals association between dRNH-specific R-loops and transcriptional pausing. (**A**) Venn diagram highlighting the group of R-loop Regions (RL Regions) analysed in this figure. (**B**) A PCA plot showing the divergence in abundance between dRNH and S9.6-based approaches within the shared RL regions. The percent of variance explained by each PC is indicated on each axis. (**C**) Volcano plot showing the top differentially abundant RL regions between dRNH and S9.6-based samples. Vertical dashed line represents absolute log2 fold change of 1, horizontal dashed line represents *P* adjusted value of *P* < 1e−30. Red: RL-regions which meet both the log_2_ fold change and *P* adjusted value cut-off. Green: RL-regions which meet the log_2_ fold change cut-off alone. (**D**) A PCA feature plot showing a top differentially abundant RL region (‘RL46345’). (**E**) Genome browser image of RL46345 highlighting difference in S9.6 and dRNH signal at that site. The genome browser session for this visualization is also provided within this manuscript (see Availability). (**F**) Top dRNH and S9.6-specific hits from GO term analysis of the genes associated with the top differentially abundant RL regions. Enrichment is measured by ‘Combined Score’ such that higher indicates more strongly enriched. (**G**) Graphical illustration which depicts the Class I/II hypothesis. Class I R-loops are hypothesized to occur as a result of promoter pausing and are efficiently mapped by dRNH and, to a lesser extent, S9.6. Class II R-loops are associated with transcriptional elongation and are mapped efficiently by S9.6 alone. (**H**) Representative distribution plots showing the pause index of genes overlapping dRNH- and S9.6-specific R-loop regions. Data were derived from precision run on (PRO) sequencing in three cell lines (HEK293, HeLa, K562) for which both dRNH and S9.6 R-loop mapping data were available. (**I**) Representative genome browser image depicting a top dRNH-specific RL region (RL12840) which occurs at a bidirectional promoter for two of the most transcriptionally paused genes, ATG3 and SLC35A5. PRO-Seq in HEK293, HeLa, and K562 is displayed alongside dRNH and S9.6 consensus tracks. P values generated via Wilcoxon rank sum test (minimum displayable *P* value is 2.22e−16).

To address these questions, we analysed differential R-loop abundance for each high-confidence sample within the 9950 R-loop regions (RL regions) found in both S9.6 and dRNH consensus sites (Figure 5A). We applied principal component analysis (PCA) and found that dRNH and S9.6 samples diverge with respect to RL region abundance even though the sites analysed here represent sites detected by both approaches (Figure 5B), indicating our ability to detect meaningful differences between them. We also repeated the PCA without quality control filters and we observed a clear separation of POS:POS (‘true positive’) and NEG:POS (‘false positive’) samples along PC2, highlighting the utility of the QC workflow we describe in this work (Supplementary Figure S5A). However, we also noticed that PC1 still cleanly separates dRNH and S9.6 samples even with low quality samples included, further highlighting the differences between these mapping approaches (Supplementary Figure S5A).

Differential abundance analysis led to the identification of RL regions for which the S9.6-derived and dRNH-derived signal intensity were discordant (Figure 5C) (see **Availability**). We then visualized the top differentially abundant RL region (RL46345) (Figure 5D, E), which reinforced the clear differences in signal intensity between dRNH- and S9.6-based R-loop mapping at that site.

One potential confounding factor in this analysis was the disparity in number of samples and studies between S9.6 (191 samples; 31 studies) and dRNH (56 samples; nine studies). To address this concern, we randomly downsampled the S9.6 dataset to match the dRNH dataset, and then we repeated the differential analysis. Of note, due to previously observed batch effects between R-loop mapping studies (16), we randomly downsampled by *study* and then by *sample* until the S9.6 dataset had the same number of studies as dRNH (9 studies) and the same number of samples or less (∼56 samples). From comparing the downsampled results to the full dataset, we found they agreed well (Pearson R: 0.95) (Supplementary Figure S5B). To validate that this result was not due to random chance during downsampling, we performed 10 random downsampling permutations and calculated the correlation across each. Like the initial finding, all permuted results showed high correlation with the full dataset and with one another (Supplementary Figure S5C). Moreover, even when we tried downsampling by sample number alone without considering the number of studies, this correlation was similarly strong (Not Shown). Taken together, these findings support the robustness of the differential R-loop abundance results.

We also wanted to address the unlikely possibility that dRNH- and S9.6-specific R-loops might coincide with genes belonging to specific biological programs. Therefore, we identified the genes overlapping the top 1000 dRNH- and S9.6-specific differentially abundant R-loops and performed GO term analysis. Unexpectedly, we found that, unlike dRNH, S9.6-based mapping methods detected R-loops in genes related to translation (Figure 5F). Conversely, dRNH-based methods detected R-loops in genes related to endoplasmic reticulum stress (Figure 5F). While these results are intriguing, it is still unclear why dRNH or S9.6 map R-loops more efficiently in genes belonging to some biological programs, so these results should be considered with caution.

The Class I/II hypothesis predicts that dRNH maps R-loops which arise from promoter–proximal pausing with high specificity compared to S9.6 (Figure 5G) (13). To address this hypothesis, we identified the genes overlapping dRNH/S9.6-specific R-loops and calculated their pausing index (58) using publicly available precision run-on (PRO) sequencing data. To improve the accuracy of the analysis, we specifically chose to utilize PRO-Seq from untreated cell lines found in both the dRNH- and S.6-based R-loop mapping datasets (HEK293, HeLa and K562) (see Availability). Of note, we filtered the data to only include R-loop regions which overlap with known transcription start sites (TSS). We observed that dRNH-specific genes show significantly greater pausing compared to S9.6-specific genes in all three cell lines (Figure 5H). This effect is exemplified by RL12840, one of the top dRNH-specific RL regions, which occurs at a bidirectional promoter for two of the top paused genes, ATG3 and SLC35A5 (Figure 5I). To further validate this finding, we mined public PRO-Seq datasets for another nine cell lines and repeated the analysis (Supplementary Figure S5D). As expected, we found in each cell line that dRNH-specific R-loops coincided with genes having a significantly higher pause index than those which overlap with S9.6-specific R-loops (Supplementary Figure S5D).

Taken together, these results further support the soundness of the Class I/II hypothesis as dRNH displays a strong preference for transcriptionally paused genes compared to S9.6. Moreover, they suggest intriguing differences between dRNH-based and S9.6-based R-loop mapping in several gene programs.

### dRNH-based mapping uniquely detect smaller R-loops associated with distal enhancers

**Figure 6.**
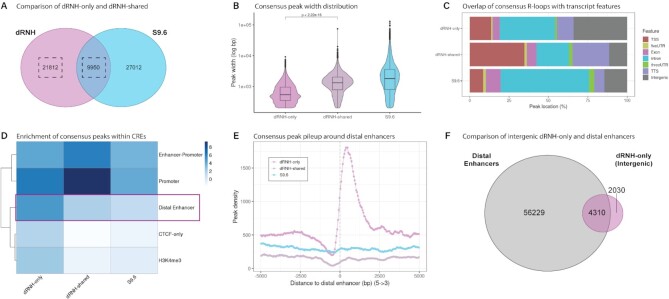
dRNH uniquely maps shorter R-loops that are enriched within distal enhancer regions. (**A**) Venn diagram highlighting the group of R-loop Regions analysed in this figure. (**B**) Stacked bar chart showing the distribution of transcript (tx) features within dRNH-only (dRNH consensus peaks not mapped by S9.6), dRNH-shared (dRNH consensus peaks which overlap with S9.6 consensus peaks), and S9.6 consensus peaks. (**C**) Distribution of peak widths (log scaled) across groups. *P* value from Wilcoxon rank sum test (‘2.22e-16’ is the minimum displayable value). (**D**) Heatmap depicting the enrichment of peaks within ENCODE-SCREEN predicted cis-regulatory elements (CREs). Values are the log_2_-transformed odds ratio from Fisher's exact test. (**E**) Line-plot showing pileup of consensus peaks around distal enhancers (GeneHancer database). (**F**) Venn diagram showing the overlap between intergenic dRNH-only peaks and distal enhancers.

From the above analysis, we found support for the Class I/II hypothesis by examining the genic distribution of dRNH and S9.6 consensus R-loops (Figure 4H-K) and the association of dRNH-specific R-loops with transcriptional pausing (Figure 5H-I, Supplementary Figure S5D). However, the Class I/II hypothesis is insufficient to fully explain the 16.4Mb of dRNH consensus sites which S9.6-based mapping does not detect (occupies 11.8% of RL Region covered genome; 0.51% of total human genome) (Figure 4F, J), especially those which occur in intergenic regions (Figure 4K). This suggested that there may be additional subclasses of R-loops which we could defined from this analysis. To begin to identify novel subclasses of R-loops, we decided to split dRNH consensus peaks into ‘dRNH-only’ (uniquely mapped by dRNH), ‘dRNH-shared’ (dRNH peaks which overlap with an S9.6 peak) (Figure 6A) and compared them to each other alongside S9.6 consensus regions.

From a preliminary analysis of peak characteristics, we noticed that dRNH-only consensus regions are smaller (mean 754 bp) than dRNH-shared (mean 1588 bp) and S9.6 (mean 3293 bp) (Figure 6B). From analysis of the genes overlapping R-loops in each group, we were surprised to observe that dRNH-only R-loops do not occur within defined gene programs, unlike dRNH-shared and S9.6 R-loops (Supplementary Figure S6A).

The smaller size and lack of distinct gene set enrichment within dRNH-only peaks suggested that they might be coinciding with specific genomic features. From feature analysis, we observed that while dRNH-shared peaks display strong enrichment in TSS and TTS regions, dRNH-only peaks are strongly over-represented in intergenic regions (∼1/3 of all peaks) (Figure 6C). This led us to suspect that dRNH may be uncovering intergenic R-loops which coincide with cis-regulatory elements (CREs).

To assess the relationship between dRNH-only R-loops and CREs, we analysed their enrichment within the predicted CRE database provided by ENCODE-SCREEN (Figure 6D). In a recent study, *Yan et al* demonstrated that MapR (a dRNH-based technique) detects R-loops at predicted enhancers, a type of CRE (12). Likewise, we also observed a strong enrichment of dRNH peaks within enhancers (Supplementary Figure S6B, C). However, we also found strong enrichment of *dRNH-only* peaks specifically at *distal* enhancer regions (Figure 6D, E, Supplementary Figure S6D). In line with this observation, we found that ∼2/3 of intergenic dRNH-only peaks overlap with distal enhancers (Figure 6F). Moreover, we observed that dRNH peaks which overlap with distal enhancers are significantly smaller than those which do not (Supplementary Figure S6E), implying that dRNH uniquely detects smaller R-loops which form within distal enhancer regions.

### dRNH-based mapping detects eRNA R-loops associated with cell type-specific gene programs

**Figure 7.**
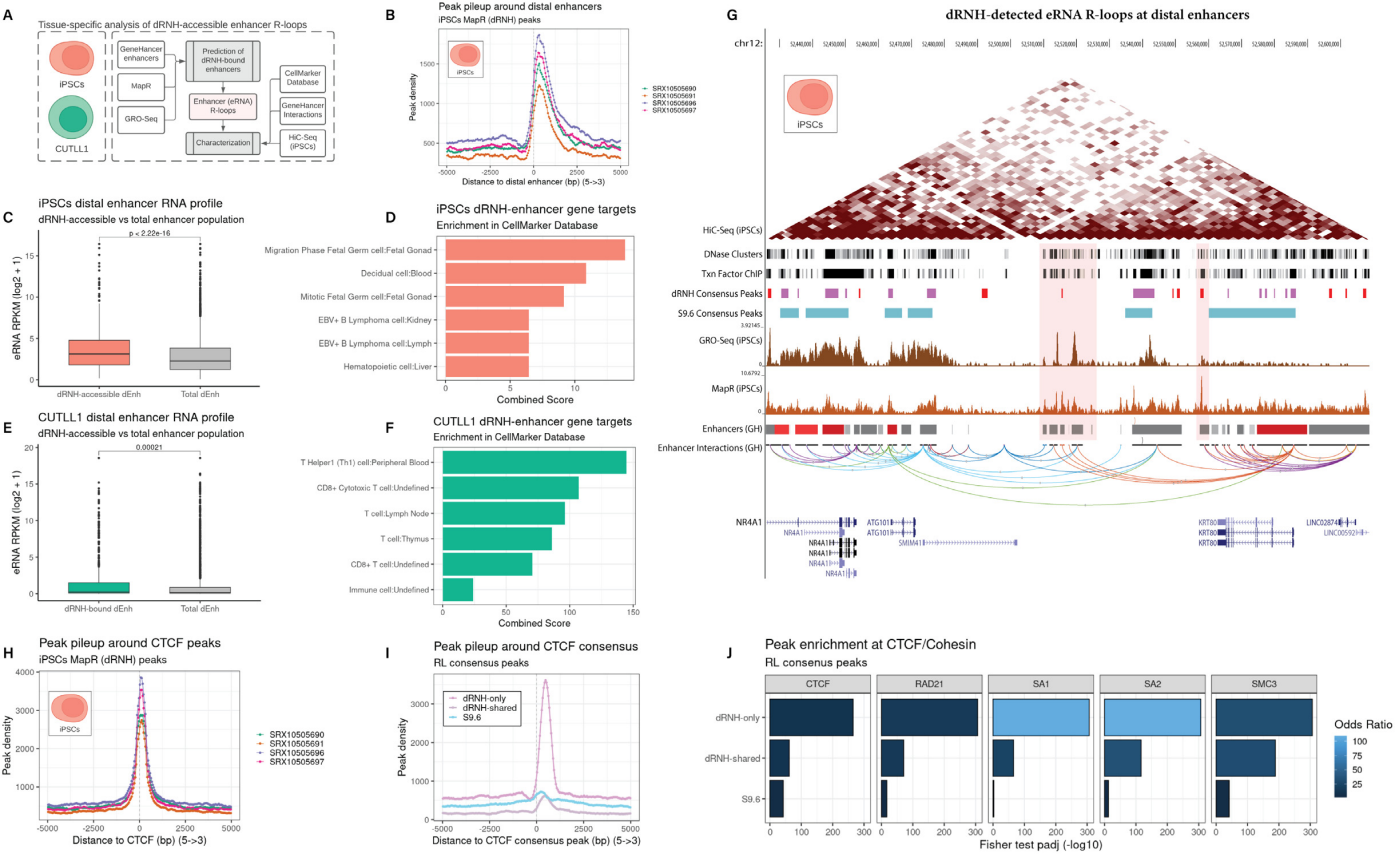
Intergenic dRNH-only R-loops occur in tissue-specific distal enhancers and co-localize with CTCF/Cohesin. (**A**) A diagram showing the workflow for tissue-specific analysis of dRNH-mapped R-loops at distal enhancers. For both iPSCs and CUTLL1 cells, tissue-specific MapR (dRNH mapping technique) and global run-on (GRO) sequencing datasets were combined with the GeneHancer enhancer database to identify distal enhancers with putative enhancer RNA (eRNA) R-loops. These enhancers were characterized using the GeneHancer interaction database and the CellMarker annotation database. Finally, they were combined with HiC-sequencing data (iPSC-only) and visualized on the genome browser. (**B**) Line plot showing the enrichment of iPSC MapR peaks (4 replicates) within distal enhancers. (**C**) For iPSC datasets, box plot showing the eRNA expression level within distal enhancers that are bound by dRNH, compared to all distal enhancers. *P* value derived from Wilcoxon rank sum test (one-sided) (minimum displayable p value is 2.22e-16). (**D**) For iPSC datasets, Bar chart depicting the enrichment of gene targets of dRNH-bound distal enhancers. Enrichment uses the CellMarker database. (**E**) Same as (**C**) but for CUTTL1 datasets. (**F**) Same as (**D**) but for CUTTL1 datasets. (**G**) Representative genome browser image showing iPSC MapR and GRO-Seq signal (mean of replicates) at multiple distal enhancers (GH: GeneHancer database) around TAD boundary. Consensus dRNH-only peaks are highlighted in the ‘dRNH Consensus Peaks’ track. The genome browser session for this visualization is also provided within this manuscript (see Availability). (**H**) Same as (B) but for iPSC CTCF peaks. (**I**) Same as (B) but for enrichment of R-loop consensus peaks around CTCF consensus peaks. (**J**) Bar chart displaying the enrichment of R-loop consensus peaks within CTCF and cohesin complex members (SA1, SA2, SMC3, RAD21). *P* adjusted value (*P*_adj_) and Odds Ratio from Fisher's exact test (maximum –log_10_*P* adjusted value: 307.7).

Consensus analysis from dozens of cell lines and tissues enabled us to identify a global enrichment of dRNH-only peaks within distal enhancers (Figure 6, Supplementary Figure S6). However, many enhancers promote cell type-specific gene programs (73). Therefore, to obtain fine-grained characterization of dRNH/enhancer relationships, we performed an analysis of dRNH R-loops in two cell lines for which we expected to observe distinctive cell type-specific enhancer activity: induced pluripotent stem cells (iPSCs) and T-cell lymphoma cells (CUTTL1) (Figure 7A).

As expected, we found a strong enrichment of MapR (a dRNH-based mapping method) peak pileup within distal enhancers in both cell lines (Figure 7B, Supplementary Figure S7A). From overlap analysis, we found that 25.92% of intergenic CUTLL1 peaks and 40.96% of intergenic iPSC peaks overlap with distal enhancers (Supplementary Figure S7B, C).

Enhancer RNA (eRNA) is a non-coding RNA species that forms within enhancer regions (74,75) and which has been previously associated with R-loop formation at enhancers (11). Therefore, we decided to determine whether MapR-detectable R-loops coincided with eRNA production and characterize the genes which those enhancers control (Figure 7C–F). As expected, we observed that distal enhancers have significantly greater transcriptional activity in both iPSCs and CUTLL1 cells (Figure 7C, E). These results suggest that R-loop formation at distal enhancers is a biproduct of eRNA transcription.

To determine whether the R-loop-bound enhancers pertained to cell type-specific gene programs, we used the GeneHancer interaction database to obtain the downstream gene targets of each enhancer. We then used the CellMarker database to test for cell type-specific gene programs and, interestingly, we observed that the R-loop-bound enhancers in both CUTTL1 and iPSCs activate gene programs specific to each tissue type (Figure 7D, F).

Taken together, these results further support the existence of eRNA R-loops and suggest that they do not necessarily impede the functionality of enhancers, particularly those which are critical to defining cell identity.

### dRNH-based mapping sensitively detects R-loop formation in CTCF/Cohesin binding sites

In our analysis of iPSC HiC-sequencing data, we observed examples of MapR-detected R-loop formation which occurred nearby topologically-associated domain (TAD) boundaries (Figure 7G). Previous studies have implicated CTCF and members of the cohesin complex as R-loop binding factors, suggesting that this binding activity may play a role in the establishment of TAD boundaries (16,76). To address this possibility, we mined CTCF chromatin immunoprecipitation sequencing (ChIP-seq) data in iPSC and CUTLL1 cell lines. Interestingly, we observed a strong enrichment of MapR peaks in CTCF ChIP-seq sites for both iPSCs and CUTLL1 (Figure 7H, Supplementary Figure S7D). Moreover, we found that a substantial proportion of intergenic MapR peaks overlap with CTCF sites (41.2% of iPSC and 27.3% CUTLL1) (Supplementary Figure S7E-F).

Having observed a strong enrichment of CTCF binding within cell type-specific dRNH data, we decided to assess whether this enrichment also occurs in a cell type-nonspecific manner. Interestingly, we observed a stark enrichment of dRNH-only (but not dRNH-shared or S9.6) peaks within CTCF consensus peaks (Figure 7I). Moreover, we obtained consensus ChIP-seq peaks for core cohesin complex members (RAD21, SA1, SA2 and SMC3) and observed an enrichment of dRNH-only peaks within each (Figure 7J). This result suggested that a subset of R-loops co-localize with CTCF/cohesin in a cell type-nonspecific manner and that dRNH detects those R-loops with higher sensitivity than S9.6.

### dRNH-based mapping detects R-loop formation at bivalent, transcriptionally silent enhancer clusters

**Figure 8.**
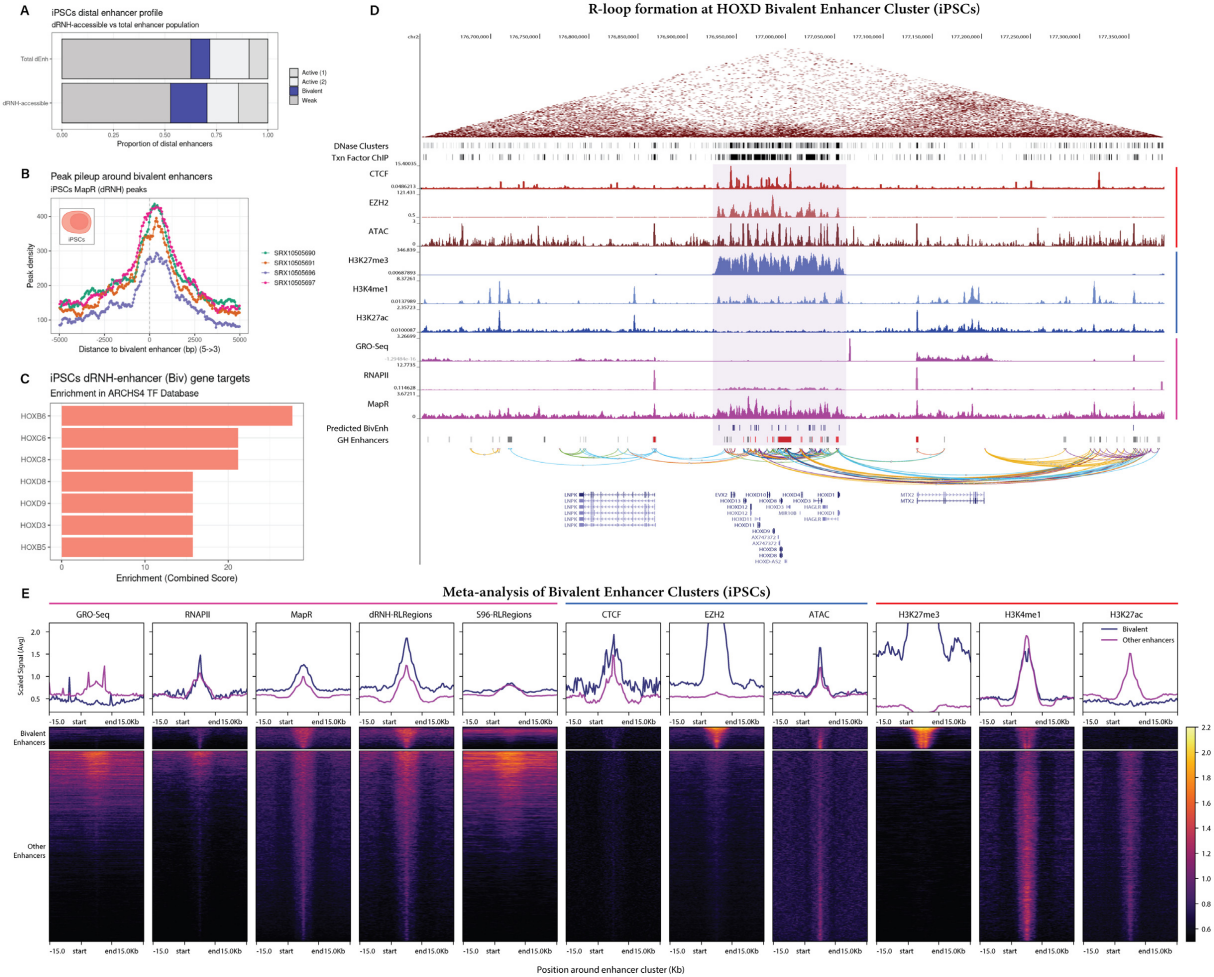
R-loops are enriched at bivalent, transcriptionally paused enhancer clusters in iPSCs. (**A**) A stacked bar chart showing the proportion of intergenic enhancers belonging to each chromHMM state. Enhancers that overlap with MapR peaks are labelled ‘dRNH-accessible’. (**B**) Line plot showing the enrichment of iPSC MapR peaks (4 replicates) within bivalent distal enhancers. (**C**) Bar chart depicting the enrichment of gene targets of dRNH-bound bivalent enhancers. Enrichment uses the ARCHS4 transcription factor co-expression database. (**D**) Representative genome browser image displaying the HOXD bivalent enhancer cluster. The genome browser session for this visualization is also provided within this manuscript (see Availability). (**E**) Tornado plots showing the pileup of signal for each track around bivalent and non-bivalent enhancer clusters. Max value in this plot is 2.2 and the minimum is 0.2.

To better understand R-loop-bound enhancers, we analysed chromHMM chromatin state data in iPSC cells (CUTLL1 data not available). Given their global association with eRNA transcription and cell type-specific gene programs, we anticipated that R-loops would be enriched at active enhancers, as was previously reported (12,77). Curiously, we found that only a quarter of R-loop-bound distal enhancers were in an active state, similar to the proportion found in the background population (Figure 8A). Moreover, we observed the greatest enrichment within bivalent enhancers at approximately 2-fold the background level (Figure 8A). From pileup analysis, we found evidence of this enrichment pattern in every available iPSC MapR replicate (Figure 8B).

Pluripotent stem cells contain a large number of bivalent enhancers which prime early developmental programs (78). To understand whether R-loop-bound bivalent enhancers were relevant to developmental programs, we performed pathway enrichment on the genes with which they interact (Figure 8C, Supplementary Figure S8A). Interestingly, we observed a marked enrichment of genes involved in programs relevant to embryonic development, including KLF2/4/5 binding targets (Supplementary Figure S8A). We also noticed a strong enrichment of homeobox (HOX) gene clusters (Figure 8C).

HOX genes are arranged co-linearly in four clusters (‘A’, ‘B’, ‘C’ and ‘D’) and act to regulate anatomical patterning during early development (79). In iPSCs and embryonic stem cells, EZH2 acts to establish repressive (H3K27me3) marks across these regions, supressing HOX transcriptional activity (79). From examination of each HOX cluster in the genome browser, we found that each contained a large (>100 kb) sized bivalent enhancer cluster marked by EZH2 binding, high R-loop levels, RNA Pol II occupancy, and low nascent transcription (Figure 8D, Supplementary Figure S8B; HOXB/C not shown), a pattern suggestive of stable transcriptional pausing. We then assessed whether this same pattern occurs across bivalent clusters genome wide. As predicted by the pattern at HOX clusters, we observed genome-wide RNA Pol II and R-loop occupancy, coinciding with a lack of nascent transcription at bivalent enhancer clusters genome-wide (Figure 8E). Interestingly, we also found that, despite the cell type-specific nature of many enhancers, dRNH consensus signal was also highly enriched at iPSC bivalent enhancer clusters (Figure 8E). As expected, however, we observed a lack of S9.6 consensus signal enrichment at these sites (Figure 8E). Taken together, these results reinforce previous reports of dRNH-mapped R-loops at bivalent enhancers (77) while offering new context that suggests these R-loops may form preferentially over transcriptionally-silent, bivalent enhancer clusters which regulate core developmental programs.

### dRNH and S9.6 differentially detect R-loops co-localizing with RNA-binding factors

While the preceding results reinforced the Class I/II hypothesis and characterized the R-loops which dRNH mapping technique uniquely detect, it remained unclear why dRNH consensus sites only cover 26.3% of the RL-region occupied genome, compared to 87.9% for S9.6 (Figure 4J). It was also unclear why S9.6 maps R-loops in gene bodies with greater efficiency than dRNH (Figures 4I, K and 6C). We decided to explore the possibility that these findings might relate to differences in the S9.6 and dRNH-based mapping protocols, particularly with respect to protease treatment.

S9.6-based mapping techniques, such as DRIP-Seq, rely upon immunoprecipitation of R-loops within proteinase-treated chromatin (2). MapR and R-ChIP, the two dominant dRNH modalities, both involve mapping of R-loops in the context of native chromatin (12,80). As such, the presence of RNA- and/or DNA-binding proteins could be one explanation for the increase in gene-body R-loop binding in S9.6 compared to dRNH. To address this possibility, we identified R-loops mapped uniquely by dRNH or S9.6, split by gene body or intergenic localization, and calculated their enrichment within experimentally determined binding sites of chromatin-binding transcription factors (ChIP) and RNA-binding factors (eCLiP) (Supplementary Figure S8C). As expected, we observed an elevated enrichment of ChIP factor binding within dRNH-only peaks and eCLiP factor binding within S9.6-only peaks (Supplementary Figure S8C). Interestingly, we also observed that there was a robust increase (*P* < 2.1E−7) in eCLiP enrichment for S9.6-only genic peaks compared to intergenic peaks. This result aligned with our prior expectations, given that most RNA transcription occurs within gene bodies. However, in dRNH-only peaks, the difference between genic and intergenic enrichment was marginal (*P* = 0.048), suggesting that dRNH may bind preferentially to R-loops that lack RNA binding factor occupancy, regardless of genic localization. Of note, a recent study by Wang *et al.* utilized *ex vivo* and *in vivo* mapping with S9.6 and dRNH and demonstrated these R-loop sensors map R-loops similarly when analysed under the same experimental conditions (81). Taken together with the findings of our present study, this suggests that dRNH modalities fail to efficiently map gene body R-loops *in vivo* due to the presence of RNA binding proteins. It remains for future studies to evaluate this hypothesis directly.

### R-loop conservation analysis reveals differences between constitutive and variable R-loop regions

Having addressed the similarities and differences between dRNH and S9.6 consensus regions, we next sought to investigate whether R-loop region (RL region) conservation across samples could highlight additional R-loop subtypes, regardless of whether those RL regions derive from S9.6 or dRNH-based mapping samples. For these experiments, we subdivided RL regions to identify those which are more ‘constitutive’ (more conserved across independent samples) or ‘variable’ (less conserved across independent samples). To accomplish this, we calculated RL region conservation percentages, defined as the proportion of high-confidence R-loop mapping samples that identify each RL region.

Prior to our main analysis, we wanted to ensure that dRNH and S9.6-specific biases would not dominate the conservation analysis. Therefore, we calculated the conservation percentage distributions for S9.6 and dRNH consensus peaks separately, finding that they tended to be similar, with a noticeable increase in conservation levels within sites found by both dRNH and S9.6 (Supplementary Figure S9A). Comfortable with this result, we proceeded with the union RL region set for the remainder of this analysis.

**Figure 9.**
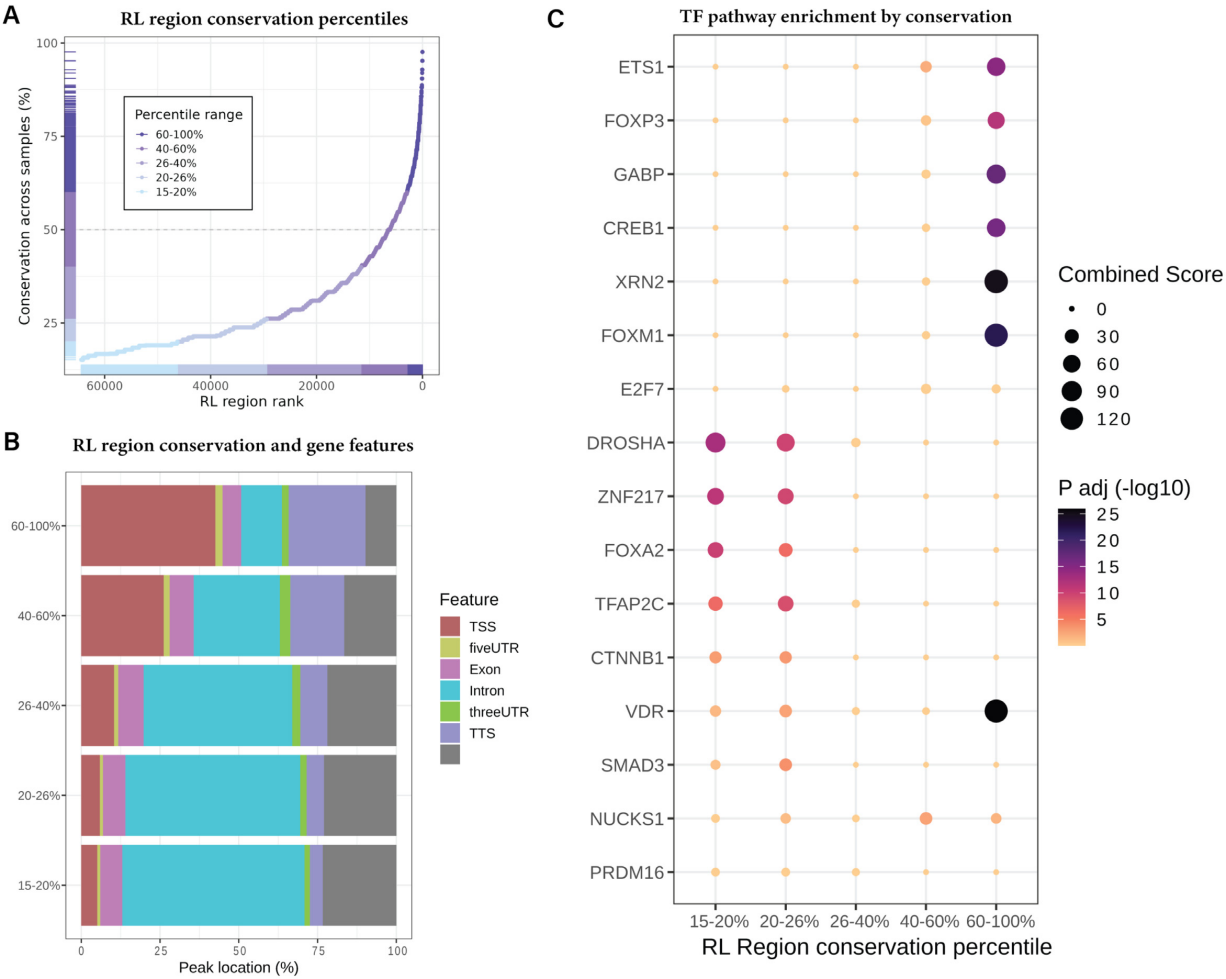
R-loop region conservation analysis. (**A**) Conservation rank plot showing all RL regions ordered by the percent of high-confidence samples in which they are found and binned by percentile ranges. (**B**) Annotation plot showing the gene features which overlap RL regions from various conservation percentile ranges. (**C**) Pathway enrichment plot showing the significance (via P adjusted value) and effect size (via Combined Score) of the ‘ChEA 2016’ transcription factor pathway enrichment from the genes overlapping RL regions within each percentile.

We examined the distribution of conservation percentages across all RL regions and assigned them to bins for discrete analysis (Figure 9A). Next, we examined the distribution of gene features overlapping the RL regions in each conservation bin. We found a strong association between high R-loop conservation and statistical enrichment within the transcription start site and transcription termination site of genes (Figure 9B). Moreover, we saw a stronger enrichment of intron and intergenic features in variable (‘15−20%’) RL regions (Figure 9B). One explanation for this observation is that more constitutive RL regions might associate with constitutively expressed ‘housekeeping genes’ (82), while variable RL regions might overlap with more cell-type-specific genes. As expected, we observed a greater enrichment of housekeeping genes in more highly conserved bins (Supplementary Figure S9B). However, unexpectedly, the greatest enrichment was in the 40-60% conservation range and not the 60–100% range (Supplementary Figure S9B). This intriguing result led us to consider the possibility that conservation bins may represent biologically distinct R-loop programs that are partially independent from active gene expression, as we previously observed in the context of iPSC MapR signal at bivalent enhancers (Figure 8E).

To better understand the biological significance of more constitutive/variable RL regions, we performed pathway enrichment on the genes overlapping the R-loops in each conservation bin. From an analysis of transcription factor binding pathways, we found stark divergence between RL regions conservation bins (Figure 9C). The strongest hit from the top conserved bin was the ‘XRN2’ target gene set. Notably, *XRN2* has been associated with R-loops in previous studies, particularly with respect to transcription termination (65). Conversely, the strongest hit from variable RL regions was the ‘DROSHA’ target gene set. *DROSHA* is also a factor that has been previously studied in the context of R-loops (83). Moreover, we found *EZH2*, a core member of the polycomb repressive complex 2 (PRC2), was also associated with variable RL regions. PRC2 has been previously associated with R-loops in development-specific genes (84,85) and we observed strong bivalent enhancer-specific enrichment of R-loops around EZH2 sites (Figure 8E). In that context, this finding reinforces the notion that cell type-specific R-loops may arise both in enhancers and in the genes which control them. Curiously, we also observed the significant enrichment of the ‘VDR’ target gene set in both more constitutive and more variable RL regions, but not in the bins between them (Figure 9C). VDR, the vitamin D receptor, has not previously been associated with R-loop biology to our knowledge, further indicating the utility of this mode of analysis for revealing otherwise unknown associations within R-loop biology.

Having demonstrated the biological relevance of distinguishing between more constitutive/variable RL regions, we proceeded to examine the KEGG and MSigDB pathways enriched within these groups. The strongest hit for the top conserved RL region bin was ‘Myc Targets V1’, an MSigDB Hallmark gene set of genes which are regulated by MYC (86). We suspected that this result might be due to the role of MYC in promoting housekeeping genes (87), genes involved in proliferation (88), and/or those involved in RNA processing (89). Curiously, we noticed the enrichment of ‘G2-M Checkpoint’ in more constitutive RL regions and ‘Mitotic Spindle’ in more variable RL regions (Supplementary Figure S9C), further reinforcing the connection with cell cycle. Moreover, we also observed the enrichment of ‘Spliceosome’ and ‘RNA transport’ in more constitutive RL regions (Supplementary Figure S9D), again highlighting a set of genes MYC regulates. While this demonstrates that MYC regulated genes have highly conserved R-loops, the function of those R-loops remains unknown. Though weaker than the more constitutive enrichment results, we also observed enrichment of the ‘Adherens junction’ pathway in the variable RL regions (Supplementary Figure S7D).

Taken together, these results demonstrate that R-loops span a range of conservation levels, with many being more constitutive and others being more variable across samples. They also reveal that more constitutive and more variable R-loops associate with divergent genomic features and gene programs, indicating they could represent biologically meaningful and programmatic subclasses of R-loops which require further exploration.

## DISCUSSION

The role of R-loops in physiology and pathology remains a topic of much study at present (3). Outstanding questions concern the types of R-loops which exist, their prevalence, their degree of conservation, their associations with epigenomic factors and processes, and the differences between various R-loop mapping approaches. Many of these questions cannot be answered by a single dataset, but instead require the meta-analysis of hundreds of samples representing diverse biological and technical conditions. In the present study, we reprocessed and standardized 810 publicly available R-loop mapping datasets, developing the largest R-loop data resource to date. From these data, we developed a new and highly accurate method of quality control and implemented it to identify high-confidence R-loop mapping samples suitable for meta-analysis. From this analysis, we defined R-loop consensus sites and elucidated the differences between S9.6 and catalytically dead RNase H1 (dRNH) mapping approaches with respect to R-loop size and locality. We then revealed the formation of dRNH-detectable R-loops across transcriptionally-silent enhancer clusters in pluripotent stem cells. Finally, we performed a conservation analysis which revealed the biological significance of more constitutive and variable R-loops.

**Figure 10.**
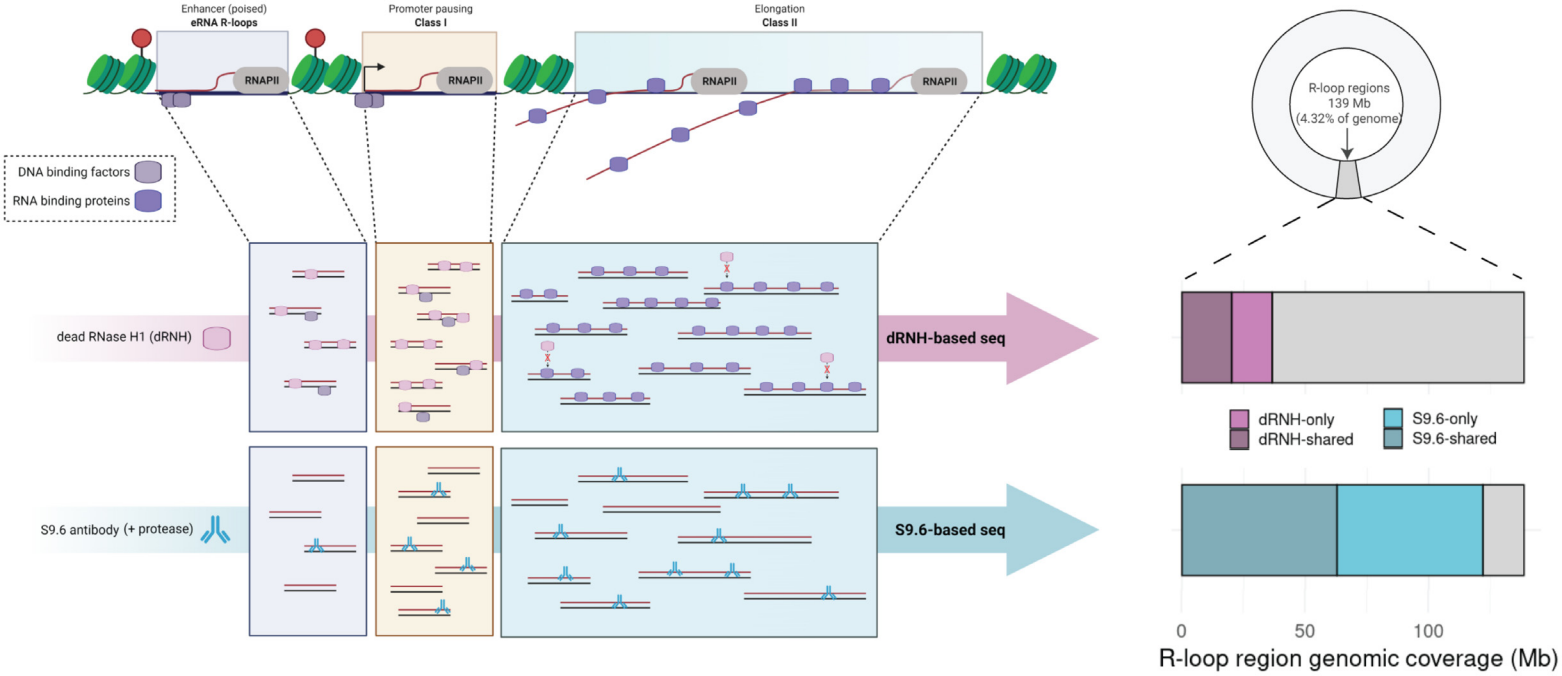
Model of differences in dRNH- and S9.6-mapped R-loops. R-loops arise as a result of transcriptional pausing at bivalent/poised enhancer regions (eRNA R-loops). They also result from promoter-proximal pausing (Class I R-loops) and transcriptional elongation (Class II R-loops). Catalytically dead RNase H1 (dRNH) efficiently maps shorter R-loops in enhancer and promoter regions, but not within gene bodies. This is ostensibly due to occupancy of Class II R-loops with RNA binding proteins (RBPs) and/or a preference of dRNH for shorter R-loops. S9.6-based mapping methods involve a protease treatment which removes RBPs, and this may explain why they map larger R-loops across the gene body. These differences are reflected in the proportion of the R-loop-forming genome (139 mega bases, ‘Mb’) which dRNH occupies (36.4 Mb) compared to S9.6 (121.8 Mb).

The results of this work supports an extension of the Class I/II model of *Castillo-Guzman and Chedin* (13) in which dRNH maps a subset of all R-loops, particularly those associated with (a) promoter proximal pausing, (b) enhancers (particularly bivalent, transcriptionally silent enhancers), and (c) regions without RNA-binding protein occupancy (Figure 10). It will remain for future studies to evaluate the molecular mechanisms of this model directly. Moreover, these efforts led us to develop new bioinformatics software packages, ‘RLSuite,’ for the analysis of R-loop datasets. While we do not describe those software packages in detail within this study, we have made them available for public access and we plan to describe them fully in a future work (see Availability).

In the following sections, we summarize the key findings and provide additional context for their relevance to the field.

### A robust quality model reveals the prevalence of poor-quality data in the literature

Within the R-loop mapping field, no methods to assess whether an individual sample has robustly mapped R-loops currently exists. Instead, studies rely only upon quality methods which are generic to all sequencing datasets, such as FastQC (11,90). Previous meta-analyses of R-loop mapping datasets relied upon comparisons with data from the literature to indirectly gauge quality (16,17), an approach which could be confounded by systematic biases affecting large numbers of studies. From these limitations, and the previous evidence of inconsistent R-loop data quality (16,17), we identified a clear need for a new R-loop mapping QC method that could benefit future researchers while also revealing the prevalence of high- and low-quality R-loop data within the field.

R-loop forming sequences (RLFS) are genomic regions which show favourability for R-loop formation (18,61–64). They are computationally predicted from genomic sequence alone and show consistency with R-loop mapping data and with other computational prediction approaches, such as *SkewR* (G or C skew prediction) (1,2,18). We calculated RLFS and found that they agreed well with the R-loop mapping data we reprocessed (Figure 1), indicating their utility as the basis for our quality control methodology. We then developed a quality model based on ensemble learning which uses the outputs from RLFS analysis to classify samples as ‘POS’ (robust R-loop mapping) or ‘NEG’ (poor R-loop mapping). This approach demonstrated excellent accuracy (Supplementary Figure S2E-F), along with both internal and external validity (Figures 2 and 3, Supplementary Figure S4). Unexpectedly, we found many samples (24.5%) with mismatches between the expected label (from sample metadata) and the predicted label from our quality model (Figure 2). Taken together, these results reveal the extent of low quality R-loop data within published datasets, indicating that the approach described here will be of great benefit to future studies. Of note, we provide access to the method described here via the RLSeq R package (see Availability).

### R-loop consensus analysis reveals the sites of robust R-loop formation

While it has been proposed that roughly 3–13% of the genome may contain R-loops, these findings were derived from an analysis of peaks from a limited number of S9.6-based R-loop mapping samples (66,70,71). From our analysis, we found stark discrepancies in the number of peaks called between studies and between R-loop mapping modalities (Supplementary Figure S1B). These findings indicate that efforts to faithfully assess R-loop locations and prevalence require a multi-study and multi-modality meta-analysis. From our meta-analysis, we identified robust sites of consensus R-loop formation, ‘R-loop regions’ (RL regions), which cover 4.32% of the human genome. These RL regions reveal the locations of R-loop formation and provide a discrete set of genomic annotations for future studies to use in the analysis of their R-loop mapping data.

### dRNH and S9.6-based mapping approaches differentially detect Class I/II R-loops in distinctive biological programs

*Castillo-Guzman and Chedin* recently proposed two classes of R-loops: ‘Class I’ and ‘Class II.’ ‘Class I’ refers to R-loops which result from promoter-proximal pausing, and ‘Class II’ refers to R-loops which result from transcriptional elongation (13). They hypothesized that dRNH-based mapping approaches may preferentially detect Class I R-loops whereas S9.6-based approaches map Class II R-loops with higher efficiency (13). However, no bioinformatics analysis had yet evaluated these R-loop classes genome wide.

From our analysis of dRNH and S9.6 consensus sites (Figures 4 and 5), we found that S9.6-based R-loop mapping tended to produce larger peaks which cover a greater proportion of the genome, compared to dRNH. We also found that dRNH-mapped R-loops tend to localize preferentially to the TSS (Figure 4H, I, K), consistent with the Class I/II hypothesis. Our subsequent analysis demonstrated that dRNH-specific R-loop regions occur in genes which have a significantly higher pausing index (Figure 5H, Supplementary Figure S5D).

Altogether, these findings support the Class I/II hypothesis. Moreover, they support the idea that dRNH-based mapping approaches may not be capable of accessing all R-loops which are detectable by S9.6-based methods.

### dRNH mapping sensitively detects smaller R-loops which occur in distal enhancer regions

While the Class I/II hypothesis is sufficient to explain R-loops detected exclusively by S9.6-based modalities, it does not fully explain the 11.8% of R-loop region genomic coverage uniquely mapped by dRNH modalities (Figure 6A). From analysis of these ‘dRNH-only’ regions, we found that they are significantly smaller than the dRNH consensus regions that co-localize with S9.6 (‘dRNH-shared’) (Figure 6B), raising the possibility that dRNH modalities can sensitively detect a subset of smaller R-loops that S9.6 modalities cannot efficiently detect. Subsequent analysis found that dRNH maps R-loops which form in enhancers with greater specificity than S9.6 (12) (Figure 6D, Supplementary Figure S6B, C), particularly distal intergenic enhancers (Figure 6D-F, Supplementary Figure S6C, D).

Distal intergenic enhancers are often involved in setting up and controlling large-scale gene programs, such as those which arise during development (73,74,74). Interestingly, these regions are also transcribed into enhancer RNA (eRNA) (74,75), though the function of eRNA remains largely unclear. To gain greater cell type-specific insight into the enhancer R-loops mapped by dRNH, we chose to examine two cell types with defined epigenetic profiles: iPSCs and CUTLL1 (a T-cell lymphoma). In both cases, dRNH-mapped R-loops were highly enriched within distal enhancers that displayed elevated transcription, as we expected given the presence of eRNA R-loops (Figure 7B, C, E, Supplementary Figure S7A–C).

Previous reports have shown that eRNAs are smaller than most lncRNA species, being ∼350 nucleotides in length on average (91). In line with this observation, we found the putative eRNA R-loops detected by dRNH are significantly smaller than other dRNH-detected R-loops (Supplementary Figure S6E). Together, these results imply that dRNH sensitively detects small eRNA R-loops which S9.6 fails to map.

### dRNH mapping detects R-loops which co-localize with CTCF and Cohesin subunits

Cohesin is a multisubunit complex which coordinates with CTCF to establish topologically-associated domain (TAD) boundaries (16). Our previous work demonstrated that cohesin members SA1 and SA2 bind R-loops directly *in vitro* (16). Moreover, we utilized a 108-sample DRIP-based consensus analysis to demonstrate that SA1, SA2 and R-loops co-localize genome wide (16). However, we had not yet evaluated R-loop/cohesin colocalization in the context of intergenic R-loops, dRNH-based techniques, nor considering other cohesin subunits. Having previously observed the cell type-specific co-localization of R-loops with TAD boundaries and CTCF (Figure 7G, H, Supplementary Figure S7D–F), we decided to return to our consensus analysis and address the degree to which R-loops co-localize with CTCF/Cohesin across tissues.

We calculated the enrichment of R-loop consensus sites within consensus peaks for CTCF and cohesin complex members SA1, SA2, RAD21 and SMC3. As expected, we observed an enrichment of intergenic R-loop consensus peaks within consensus sites for CTCF and all cohesin subunits (Figure 7J). Intriguingly, however, we observed that dRNH-only peaks show a far stronger enrichment within these regions, particularly within SA1 and SA2 bound regions (Figure 7J). This result implies that dRNH-based modalities may be useful for obtaining fine-grained mapping of genome-wide R-loop/cohesin colocalization.

Taken together, these results confirm and extend our previous findings (16) by showing that R-loops co-localize with CTCF and major cohesin subunits genome wide in a tissue non-specific manner. They also suggest that dRNH-based techniques may be better-suited for measuring these interactions.

### dRNH mapping detects eRNA R-loops at transcriptionally silent, bivalent enhancers

S9.6-based mapping studies have demonstrated that R-loops are associated with both active and repressive genic chromatin states (2,92,93) and are associated with the maintenance of epigenetic memory during reprogramming (93). dRNH-based studies have previously shown R-loop formation in enhancers genome-wide (11,12). Most recently, *Wulfridge and Sarma* used a dRNH-based method (BisMapR) to demonstrate that R-loops form within active (H3K27ac + H3K4me1/3) and bivalent/poised (H3K27me3 + H3K4me1/3) enhancers (77).

*Wulfridge and Sarma* found that dRNH-mapped R-loops are most enriched within active enhancers, though this had not been examined at distal sites (77). In contrast to this finding, we observed a lack of enrichment in *active* enhancers. Instead, we found a strong and consistent enrichment within *bivalent* enhancers (Figure 8A-B). One potential reason for this discrepancy is that iPSCs (the cell type used in this analysis) have a large number of bivalent enhancers which regulate developmental gene programs, such as homeobox (HOX) genes (79). From further analysis, we observed that HOX clusters display a highly bivalent histone profile and elevated levels of R-loops (Figure 8C, D, Supplementary Figure S8B). Curiously, we observed that these regions also display RNA Pol II occupancy alongside low levels of nascent RNA transcription (Figure 8D, Supplementary Figure S8B), a pattern that suggests transcriptional pausing. Furthermore, this pattern was observed across bivalent enhancer clusters genome-wide (Figure 8E).

Multiple mechanisms have been proposed which have the potential to explain R-loop formation at these transcriptionally suppressed, bivalent enhancers. In 2020, *Alecki et al*. demonstrated in *Drosophila* that PRC2 promotes DNA duplex invasion by RNA in *trans*, leading to formation of R-loops which help to further recruit PRC1 and PRC2 to the chromatin (84). This mechanism explains the co-localization of R-loops with bivalent enhancers. However, it does not explain the abundance of RNA Pol II coincident with low nascent transcription at these sites in human iPSCs (Figure 8D, E), an observation which suggests R-loop formation in *cis* due to transcriptional pausing. Alternatively, *Skourti-Stathaki et al* demonstrated in mouse cells that, at certain genes, R-loops resulting from transcriptional pausing may help recruit polycomb proteins to repress transcription (85). While Skourti-Stathaki *et al.* described this phenomenon in the context of promoter pausing at a subset of developmental genes, it also aligns with our genome-wide observation of R-loop formation at bivalent enhancer clusters with transcriptional pausing. As a result, we propose herein an extension of the model developed by Skourti-Stathaki *et al.* to also apply at bivalent enhancers as well.

### dRNH-mapped R-loops do not coincide with RNA binding protein occupancy

The results presented herein strongly support the Class I/II hypothesis and help to partially explain the presence of dRNH-only peaks, particularly in intergenic regions. However, it is still not known why S9.6-based mapping is capable of efficiently detecting R-loops over a larger proportion of the genome compared to dRNH, or why S9.6-based mapping is more efficient in detecting R-loops which occur in gene bodies downstream of the promoter proximal pause site (Figure 4I–K). One possible explanation is that dRNH-based techniques map R-loops *in vivo* and S9.6-based techniques map R-loops *ex vivo*. This notion was recently addressed by *Wang et al* who demonstrated that S9.6 and dRNH are capable of mapping similar subsets of R-loops when both are applied in the same *ex vivo* or *in vivo* conditions (81). One possible explanation for the breadth of R-loops detected with *ex vivo* mapping strategies is their implementation of protease treatment which removes RNA- and DNA- binding proteins prior to immunoprecipitation (IP). Thus, we hypothesized that access to R-loops within gene bodies might be restricted *in vivo* by the presence of RNA binding proteins (RBPs). To assess this hypothesis directly would require a follow-up study. However, by leveraging publicly available RBP and transcription factor ChIP/eCLiP profiles, we employed an indirect, associative test.

We observed an enrichment of S9.6 consensus peaks within RBP eCLiP binding sites (Supplementary Figure S8C). We also observed a stark increase in eCLiP RBP binding within genic S9.6 consensus peaks compared to intergenic peaks, as we expected given that transcription typically occurs within genes (Supplementary Figure S8C). However, we did not observe the same phenomenon with dRNH consensus peaks (Supplementary Figure S8C). Though associative and indirect, this result implies that R-loop sensors may be excluded from sites where RBPs bind, offering a potential explanation for the inefficiency of *in vivo* R-loop mapping for detecting Class II R-loops.

### R-loop conservation levels relates to distinct biological pathways

Another outstanding question in the R-loop field relates to the dynamics of more constitutive R-loops (those which are consistently abundant across samples) and variable R-loops (those which form under specific biological conditions). To address this question, we analysed sites of R-loop formation, binned by level of conservation across samples (Figure 9, Supplementary Figure S9). From our analysis, we found a strong relationship between RL region conservation and enrichment within TSS and TTS regions of genes. Because R-loops tend to form as a result of transcription in some genes, we decided to test whether high R-loop conservation was associated with ‘housekeeping genes’, genes which are thought to be constitutively expressed in most cell types (82). Curiously, this analysis revealed that while more constitutive R-loops co-localize with many housekeeping genes, these genes were not as strongly associated with the most highly conserved RL regions (Supplementary Figure S9B). This unexpected result may indicate that while high R-loop conservation is associated with constitutive gene expression, there may be more constitutive R-loops which form more independently from gene expression. Additionally, we also revealed a striking divergence in the biological programs and pathways enriched within more constitutive/variable RL regions (Figure 9C, Supplementary Figure S9C-D). These findings indicate new and interesting directions for future R-loop studies to pursue.

### Limitations

#### Quality model training data

While the quality control method proposed in this study is both internally and externally valid (Figures 2 and 3, Supplementary Figures S2, S3A, S4), it may not be well-suited to assess the R-loop mapping techniques introduced in the future or those for which limited data is currently available. As of this writing, there are 23 different techniques for R-loop mapping, 14 of which were introduced since 2019, with seven introduced in 2021 alone (Supplementary Table S1). Many of these techniques rely upon approaches that do not use either S9.6 or dRNH, such as m6A-DIP (94) and could pose unforeseen challenges for our quality model. Moreover, several of these techniques are represented by few samples and studies, making it difficult to generalize about them. Finally, some species have few R-loop mapping samples available. To address these limitations, we have developed an online learning scheme whereby an operator can, with minimal training and software experience, rebuild the model in a matter of minutes to include new datasets. To facilitate this process, we developed a user-friendly web-browser interface for interacting with the model-building software (Supplementary Figure S2A–D). This workflow offers a convenient and automatic model building scheme that makes it easy for us to add new data as it becomes available so that our model continually improves over time.

#### Consensus analysis

We derived R-loop consensus sites from 56 dRNH and 191 S9.6 high-confidence R-loop mapping samples. This represents all the available human data which passed our quality thresholds. However, it still indicates a significant imbalance between dRNH and S9.6, leading to potential inconsistencies in the accuracy of our findings. To address the impact of this discrepancy on the results, we randomly downsampled the S9.6 dataset to match dRNH and demonstrated the stability of our differential analysis results across random downsampling permutations (Supplementary Figure S5B, C). However, there may be other unforeseen consequences of this discrepancy in sample number which our permutation testing did not address. Therefore, we have also made provisions to ensure that the process of incorporating new data is as convenient and automatic as possible. We expect this will make it easy for us to regularly update the database and, along with it, the robustness of the biological interpretation of these data.

A second caveat of this analysis was the need to select a cut-off for ‘calling’ consensus peaks. In this analysis, we chose a cut-off of 15% such that the ‘summit’ (point of highest signal) within the consensus peak is supported by at least 15% of high-confidence samples (8 samples for dRNH and 28 samples for S9.6). The choice of 15% was based on the desire to obtain ‘variable’ RL regions, while reducing the potential for spurious peaks. We chose this cut-off based on manual examination of the number and ‘noisiness’ of peaks produced at 5%, 10% and 15% thresholds (not shown). In future studies, an automated approach for selecting this cut-off should be developed.

Another caveat for the joint analysis of S9.6 and dRNH-derived data is that there are large discrepancies between the size of peaks and consensus sites between them (Figures 4G and 6B). S9.6-derived peaks and consensus sites tend to be much wider than those from dRNH (Figure 4G, Supplementary Figure S6B). This may be partially due to the prevalence of DRIP-Seq data in the S9.6 group. Because DRIP-Seq relies upon restriction enzyme digestion, it may be biased toward larger peaks. Analyses for which this poses an immediate problem are those involving enrichment in genomic features when a feature priority is used (Figures 4K and 6C) as larger peaks may overlap with more genomic features, leading to spurious over-enrichment in top-priority features. Additionally, for analyses which involve finding genes that overlap with R-loop sites, larger peaks may lead to overlaps with many more genes than shorter peaks would find. To address this issue, we forced the peaks to a uniform width centered around peak ‘summits’ (the areas of highest enrichment from peak calling) for these analyses.

#### Bisulfite samples and stranded data analysis

We excluded bisulfite samples from our analysis due to the added complexity of processing them and the small number of available samples (only 27 in total). However, these techniques should provide more accurate nucleotide-level R-loop mapping. Therefore, in future versions of the software used for this analysis, we will include methods for analysing these data types. Another limitation of this analysis is that it does not incorporate strand assignment for any of the strand-specific sequencing techniques included in the dataset. This is intentional as DRIP-Sequencing (an *unstranded* mapping method) is still the most well-represented modality and all analyses require conversion to unstranded data for compatibility with it. However, as stranded techniques gain in popularity and begin to outpace DRIP-Seq, we anticipate that future versions of the software used for processing these data will incorporate strand information. Therefore, we expect to eventually update the analyses presented in this study with stranded data to reveal higher-resolution insights into R-loop dynamics.

## CONCLUSION

In this study, we reprocessed and standardized 810 public R-loop mapping samples, introducing a novel method of quality control to assess them. From the results, we demonstrate the widespread inconsistency of dataset quality within the literature and highlight the need for future studies to adopt a quality control approach such as we provide here. Moreover, from the high-confidence R-loop samples identified herein, we proceeded to define RL regions, sites of R-loop consensus across studies. From an analysis of these regions, we revealed the stark differences between the R-loops found by dRNH and S9.6-based techniques. Furthermore, we uncovered new biologically relevant subtypes of R-loops based on their conservation across samples and indicate that programmatically different R-loops exist, more than the previously hypothesized class I/II R-loops. Taken together, this study introduces new methods and exciting future research questions for the R-loop mapping field.

## DATA AVAILABILITY

No new raw data were generated as part of this study. Processed data files generated as part of this study are provided via RLHub: https://bioconductor.org/packages/release/data/experiment/html/RLHub.html. The list of R-loop regions (RL regions) and the list of differential RL regions between S9.6 and dRNH samples are provided on Figshare: https://doi.org/10.6084/m9.figshare.16920475.v2. UCSC genome browser sessions shown in this study are also provided:

1. Figure 1B: https://genome.ucsc.edu/s/millerh1%40livemail.uthscsa.edu/NAR_2022_Fig_1B
2. Supplementary Figure S2C: https://genome.ucsc.edu/s/millerh1%40livemail.uthscsa.edu/NAR_2022_Fig_S2C
3. Figure 3A: https://genome.ucsc.edu/s/millerh1%40livemail.uthscsa.edu/NAR_2022_Fig_3A
4. Figure 4H: https://genome.ucsc.edu/s/millerh1%40livemail.uthscsa.edu/NAR_2022_Fig_4H
5. Figure 5E: https://genome.ucsc.edu/s/millerh1%40livemail.uthscsa.edu/NAR_2022_Fig_5E
6. Figure 5I: https://genome.ucsc.edu/s/millerh1%40livemail.uthscsa.edu/NAR_2022_Fig_5I
7. Figure 7G: https://genome.ucsc.edu/s/millerh1%40livemail.uthscsa.edu/NAR_2022_Fig_7G
8. Figure 8D: https://genome.ucsc.edu/s/millerh1%40livemail.uthscsa.edu/NAR_2022_Fig_8D
9. Figure S8B: https://genome.ucsc.edu/s/millerh1%40livemail.uthscsa.edu/NAR_2022_Fig_S8B

The code used in this study is provided publicly on GitHub. The repository containing the workflow for recreating all upstream data analyses is provided here: https://github.com/Bishop-Laboratory/RLBase-data. The repository containing the code used to generate all figures and tables in this manuscript is provided here: https://github.com/Bishop-Laboratory/RLoop-QC-Meta-Analysis-Miller-2022. The software developed as part of the present study includes *RLPipes* (https://anaconda.org/bioconda/rlpipes), *RLHub* (https://bioconductor.org/packages/release/data/experiment/html/RLHub.html), and *RLSeq* (https://bioconductor.org/packages/release/bioc/html/RLSeq.html).

All accessions for data used in this study are listed in Supplementary Table S1.

## Supplementary Material

gkac537_Supplemental_Files

## Notes

Present address: Alexander Bishop, Greehey Children's Cancer Research Institute, Department of Cell Systems and Anatomy, UT Health San Antonio, San Antonio, TX 78229, USA.
