# Supplementary material for: Quality-controlled R-loop meta-analysis reveals the characteristics of R-loop consensus regions
Source: Nucleic Acids Res. 2022 Jun 27;50(13):7260–86. doi: 10.1093/nar/gkac537 (PMC9303298; doi:10.1093/nar/gkac537)
Supplement: gkac537_Supplemental_Files [file gkac537_supplemental_files.zip › Resubmission_Supplemental_Figures-compressed_3.pdf]

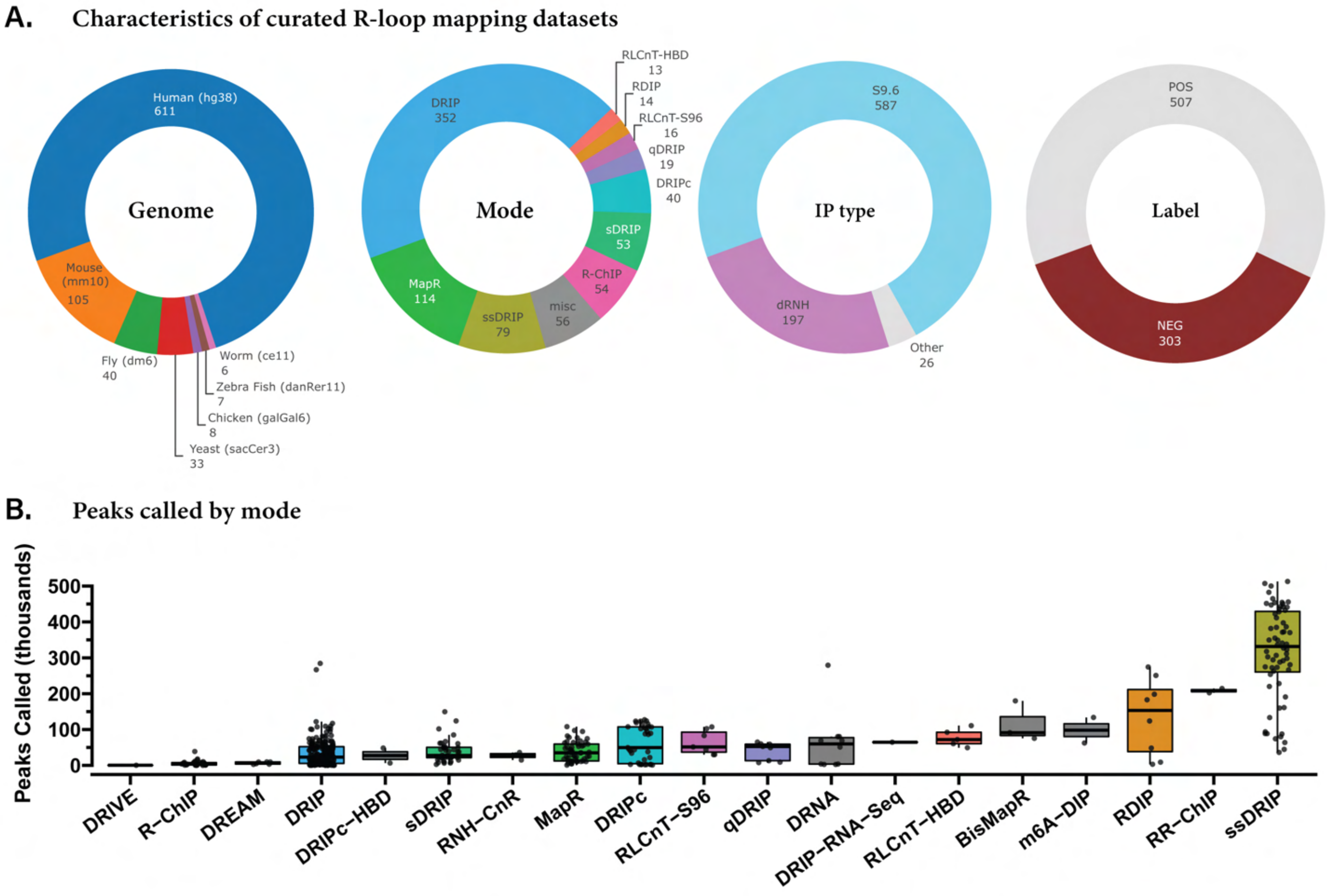

Figure S1. Overview of reprocessed R-loop mapping samples. (A) Donut charts summarizing the proportion of different genomes represented in the manually-curated datasets ("Genome"), R-loop mapping modes in the datasets ("Mode"), the proportion of immunoprecipitation types for those modes ("IP type"), and the binarized labels which correspond to the sample metadata and indicate whether a sample is expected to map R-loops ("POS") or is not expected to map R-loops ("NEG") ("Label"). (B) Box+jitter plots showing the number of peaks called in the reprocessed samples, split by mapping mode. Y axis shows the number of peaks in thousands.

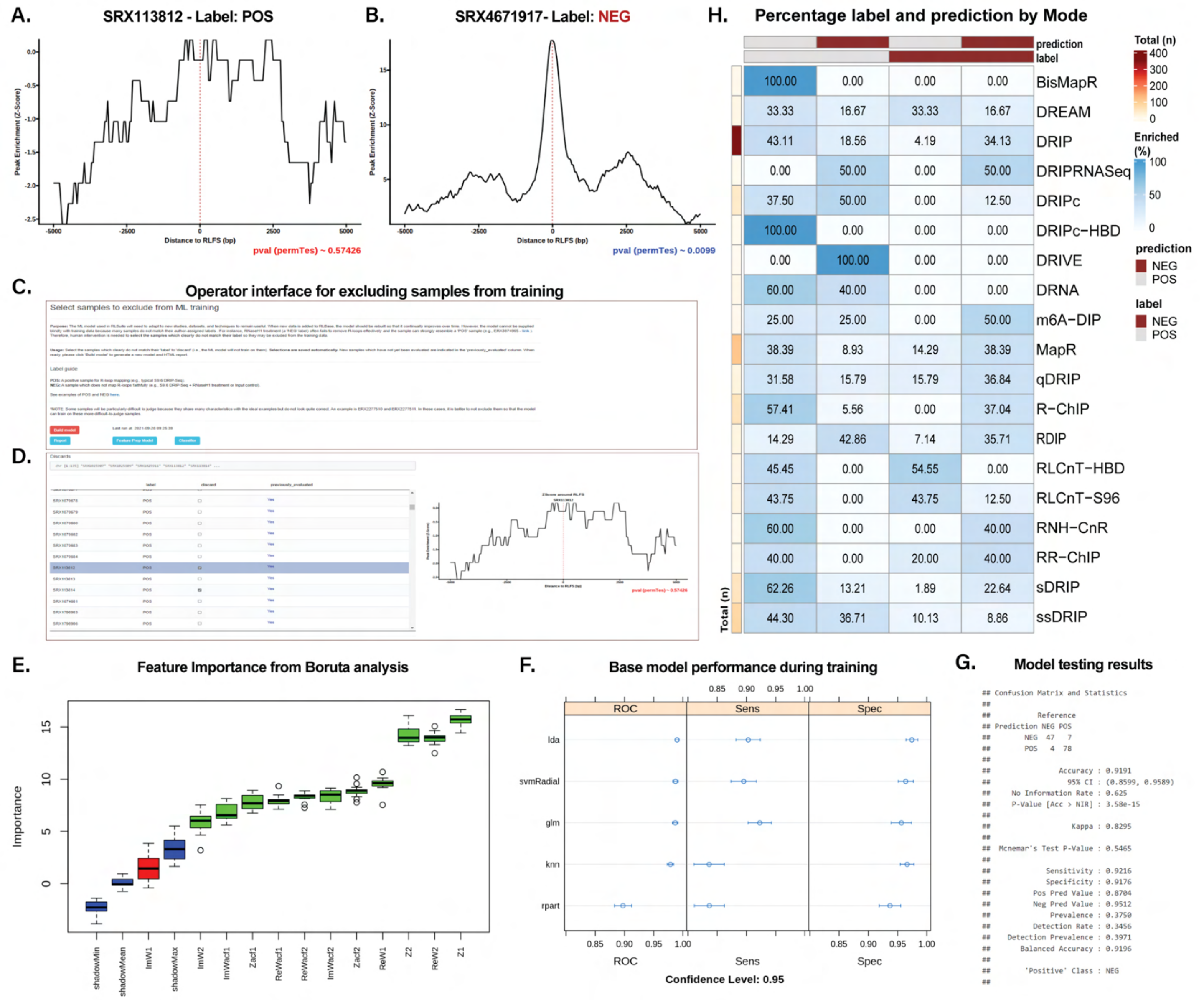

Figure S2. Model training and results. (A) Permutation testing plot showing a "discordant" RLFS analysis result (the Z score distribution resembles a negative/NEG sample, but the label is "POS"). (B) Discordant RLFS analysis results in which the permutation testing P value ($p < 0.0099$) does not match the "NEG" label and it does not appropriately represent the Z score distribution. For (A) and (B), the minimum possible p value is 0.0099. (C-D) The operator interface for semi-supervised model training. (C) Instructions and links to examples for the operator to use. The "Build Model" button will automatically launch the model building script and upload the results. This section also contains the timestamp from the last model building run. (D) A data table with one entry per sample that provides the sample label, a checkbox which is used to discard the sample from model building, and a column "previously_evaluated" which indicates whether the sample was previously seen by an operator before the last model version was built. The row selected in this table also controls which plot is shown. The plot is an RLFS Z-score distribution plot corresponding to the sample selected in the table. This plot shows a sample which was labeled "POS" (expected to map R-loops), but which an operator chose to discard as the Z score distribution did not fit the assigned label. The p value annotation relates to the p value from permutation testing (see Methods). (E) The box plot showing the feature importance of each engineered feature in the discovery set as determined by analysis with Boruta (see Methods). (F) A model evaluation plot showing the receiver operator characteristics (ROC), the sensitivity (Sens), and the specificity (Spec) for each base model in the classifier during training. Confidence intervals were obtained by repeated cross-validation (see Methods) and indicate the range of potential values for each model. (G) The model performance on the test set as directly reported by the model. This includes a confusion matrix which shows the number of correct and incorrect predictions. (H) A heatmap showing the proportion of prediction:label combinations for all samples by mode.

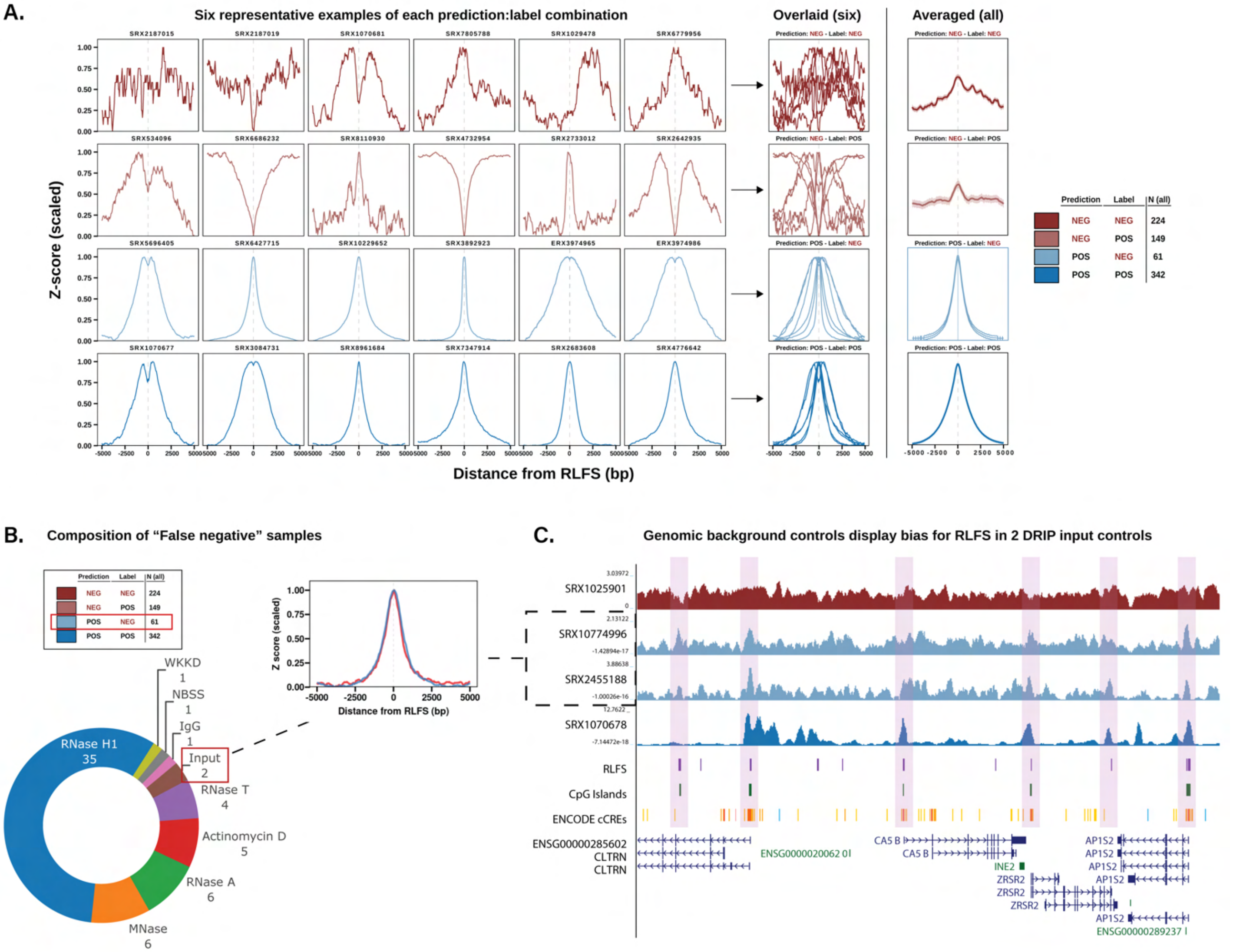

Figure S3. Extension of Figure 2. (A) The grid of figures labeled "Six representative examples of each prediction:label combination" display R-loop forming sequences (RLFS) metaplots which show the enrichment of the peaks within each sample around RLFS. The column of figures labeled "Overlaid (six)" shows the overlay of the six figures in each prediction:label group. The column labeled "Averaged (all)" shows the summarization of all data within each prediction:label group (see legend for n), calculated via loess regression. (B) Donut chart showing the composition of the "false negative" (POS:NEG) group. Results suggest that the majority (50/61; 82%) of "false negatives" result from incomplete RNase/Actinomycin D treatments. (C) Examination of the two genomic input control "false negative" results. For each, the overlaid RLFS analysis results are shown, demonstrating strong enrichment in all cases. Genome browser tracks for each along with a "true positive" (SRX1060678) and "true negative" (SRX1025901). Highlights indicate overlap of signal peaks and RLFS sites. Genome browser sessions are provided with this manuscript (see **Availability**).

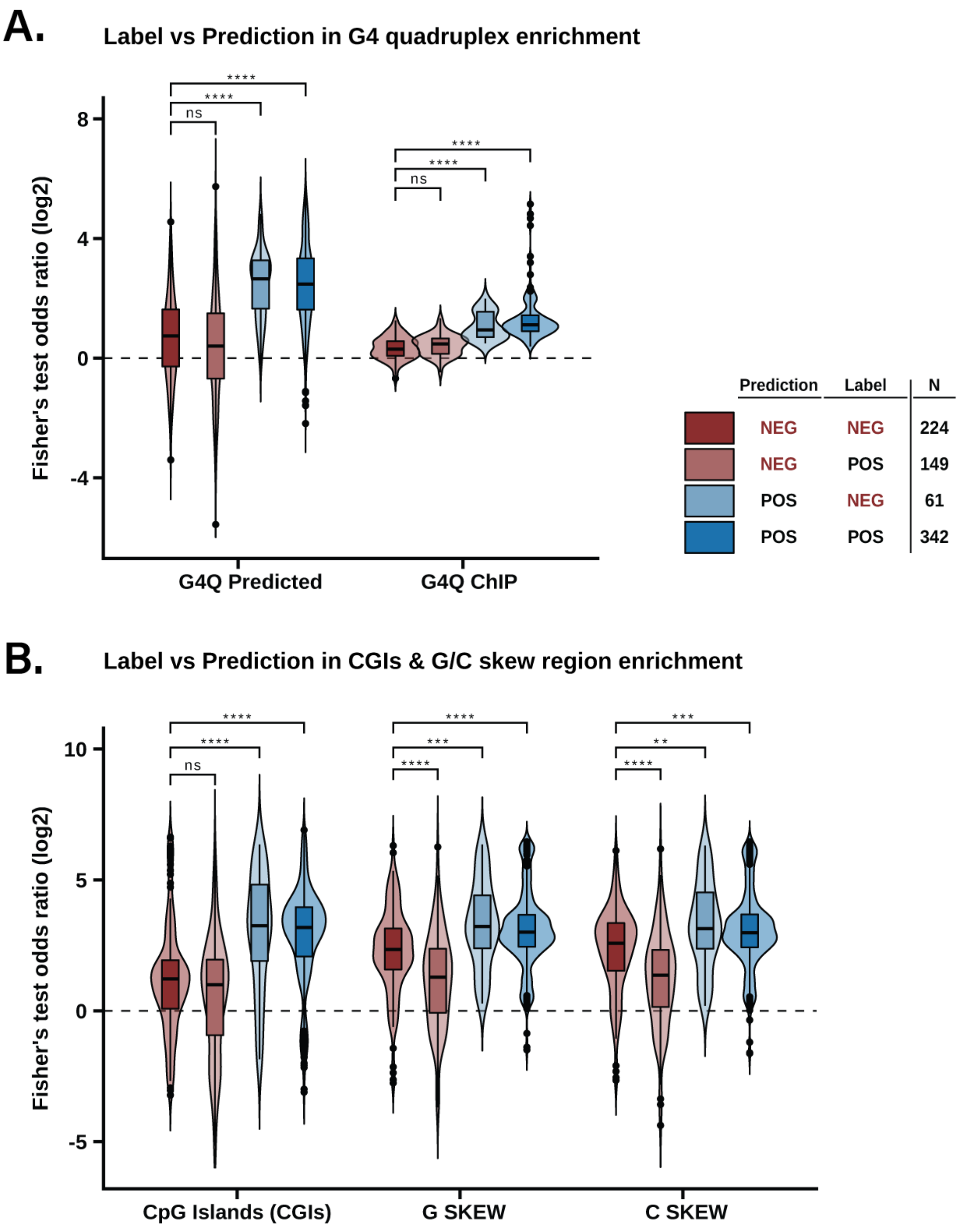

Figure S4. Extension of Figure 3. (A-B) Violin/Box plots showing the distribution of Fisher's exact test odds ratios within all reprocessed R-loop mapping samples, split by prediction:label combination, for enrichment testing within (A) G4 quadruplex sites that are computationally predicted (G4Q Predicted) or experimentally determined via ChIP sequencing (G4Q ChIP) or (B) CpG islands (CGIs) and regions of computationally predicted G or C skew. Significance was determined via the Kruskal-Wallis test followed by Dunn post-hoc with Bonferroni correction. The legend shows the number of samples within each prediction:label combination. **** - $p < .0001$; *** - $p < .001$; ** - $p < .01$; * - $p < .05$; ns – $p >= .05$.

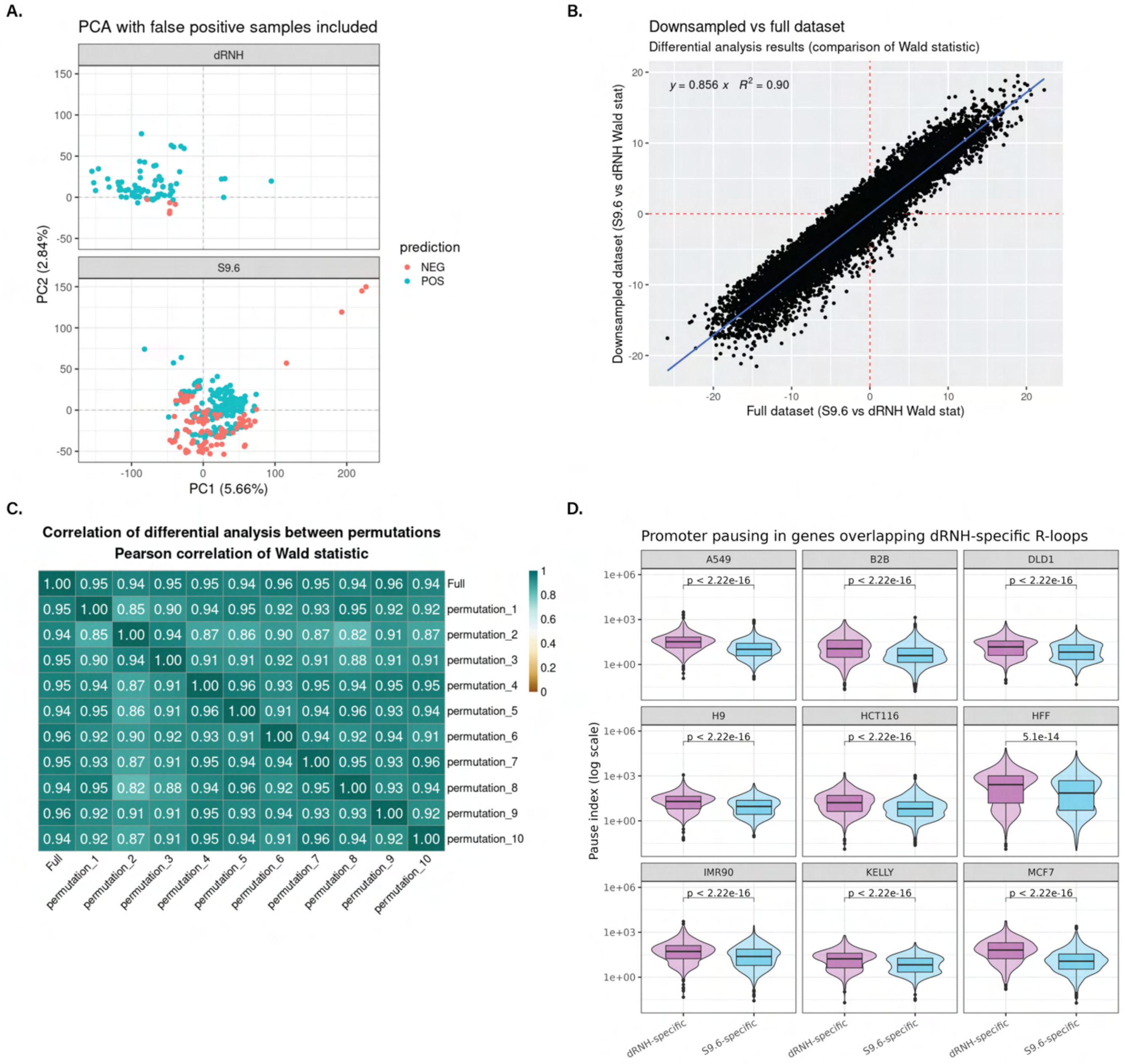

Figure S5. Extension of Figure 5 results. (A) PCA plot showing dataset without removal of “false negative” (NEG:POS prediction:label) samples. Percent of variance explained for each PC is indicated on the axis labels. (B) 4-way plot showing the correlation between dRNH vs S9.6 differential R-loop abundance (Wald statistic) in the full dataset vs one in which S9.6 was randomly down sampled. (C) Heatmap depicting the Pearson correlation of Wald statistic results between the full dataset and 10 random down sampled permutations. (D) Additional analysis of promoter pausing in additional precision run-on (PRO) sequencing datasets. Cell lines were chosen based on match with (or similarity with) cell lines represented in curated dRNH and S9.6 datasets. Pausing index was calculated for genes overlapping dRNH- and S9.6-specific RL Regions. P values generated via Wilcoxon rank sum test (minimum displayable p value is 2.22e-16).

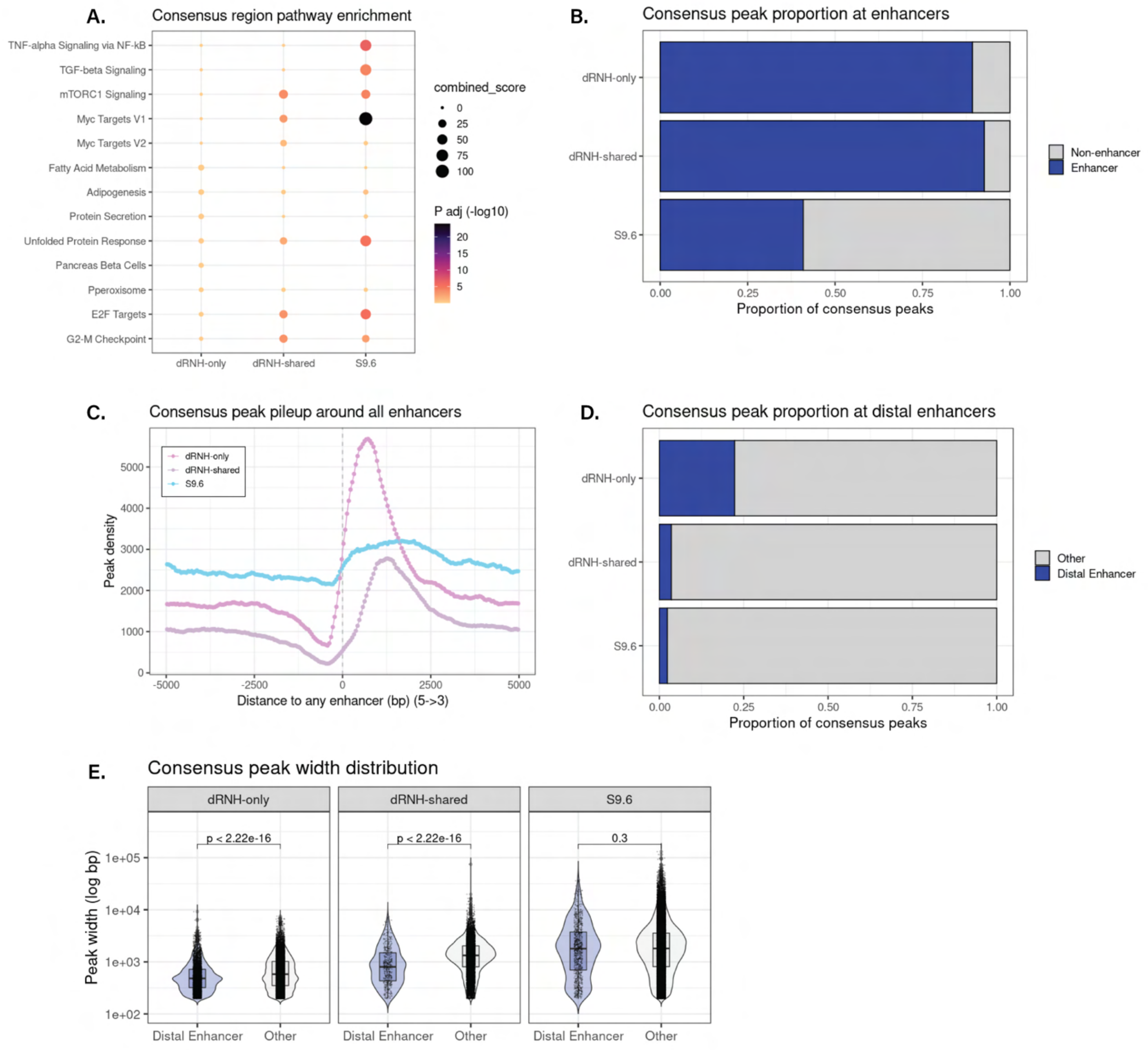

Figure S6. Extension of Figure 6 results. (A) An enrichment plot depicting the over-representation analysis results for genes overlapping the top 2000 consensus peaks from each group. Gene sets are from the MSigDB “Hallmark” gene set database. “Combined score” was capped at 100 to improve visibility of datapoints. (B) Stacked bar chart depicting the proportion of consensus peaks in each group which overlap an enhancer (GeneHancer database). (C) Meta plot showing the enrichment of consensus peaks within enhancers. (D) Same as (B) but only for distal enhancers. (E) Distribution plot showing the width of consensus peaks which overlap with distal enhancers compared to those which do not. P values from Wilcoxon rank sum test (minimum displayable p value: 2.22e-16).

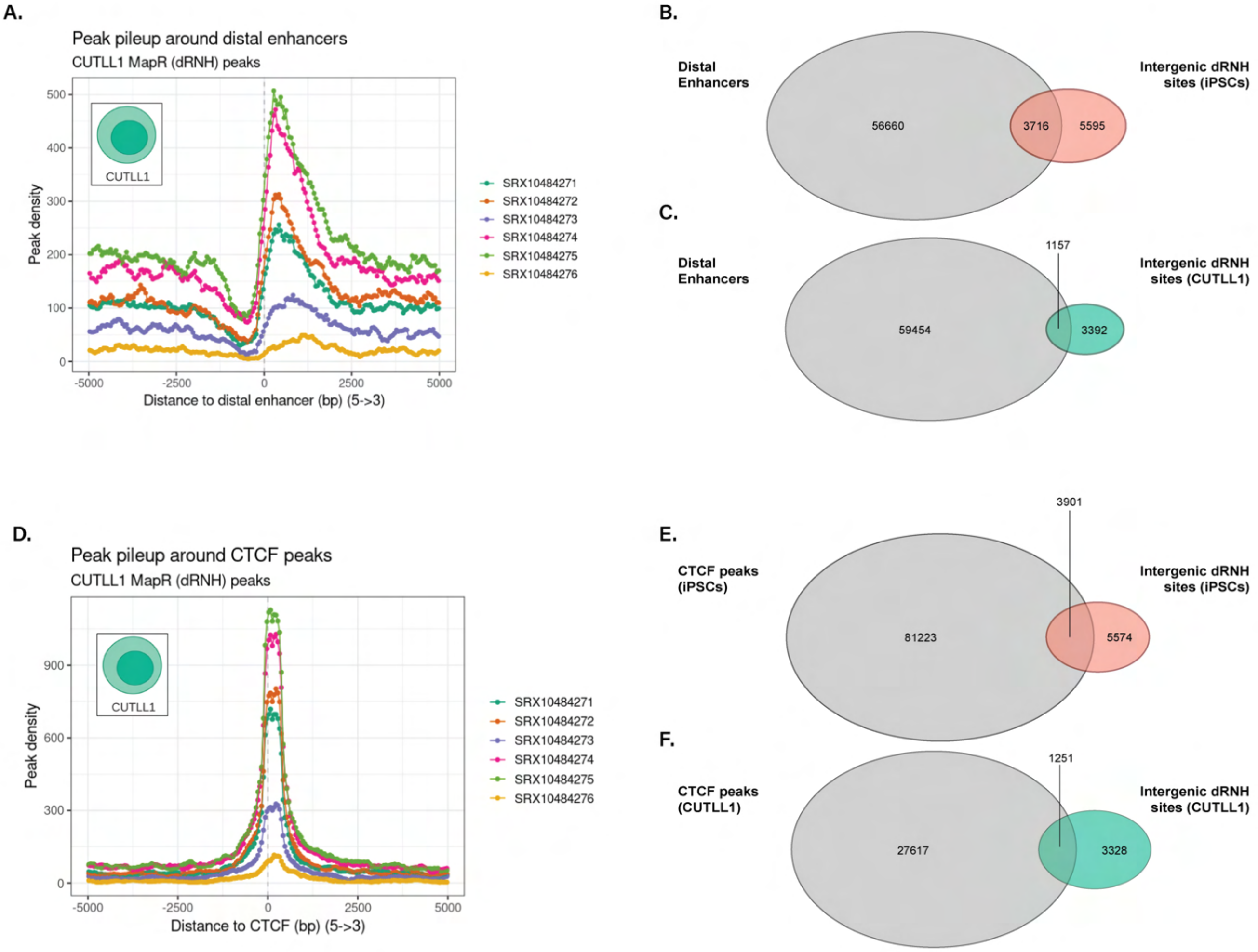

Figure S7. Extension of Figure 7 results. (A) For CUTTL1 datasets, a metaplot depicting the pileup of MapR peaks (per replicate) around distal enhancers (GeneHancer database). (B) For CUTTL1 datasets, a Venn diagram depicting the overlap of intergenic MapR peaks and distal enhancers. (C) Same as (B) but for iPSC datasets. (D) Sample as (A) but for CTCF peaks in CUTLL1 cells. (E-F) Same as (B-C) but for CTCF peaks in (E) iPSC and (F) CUTLL1 cells.

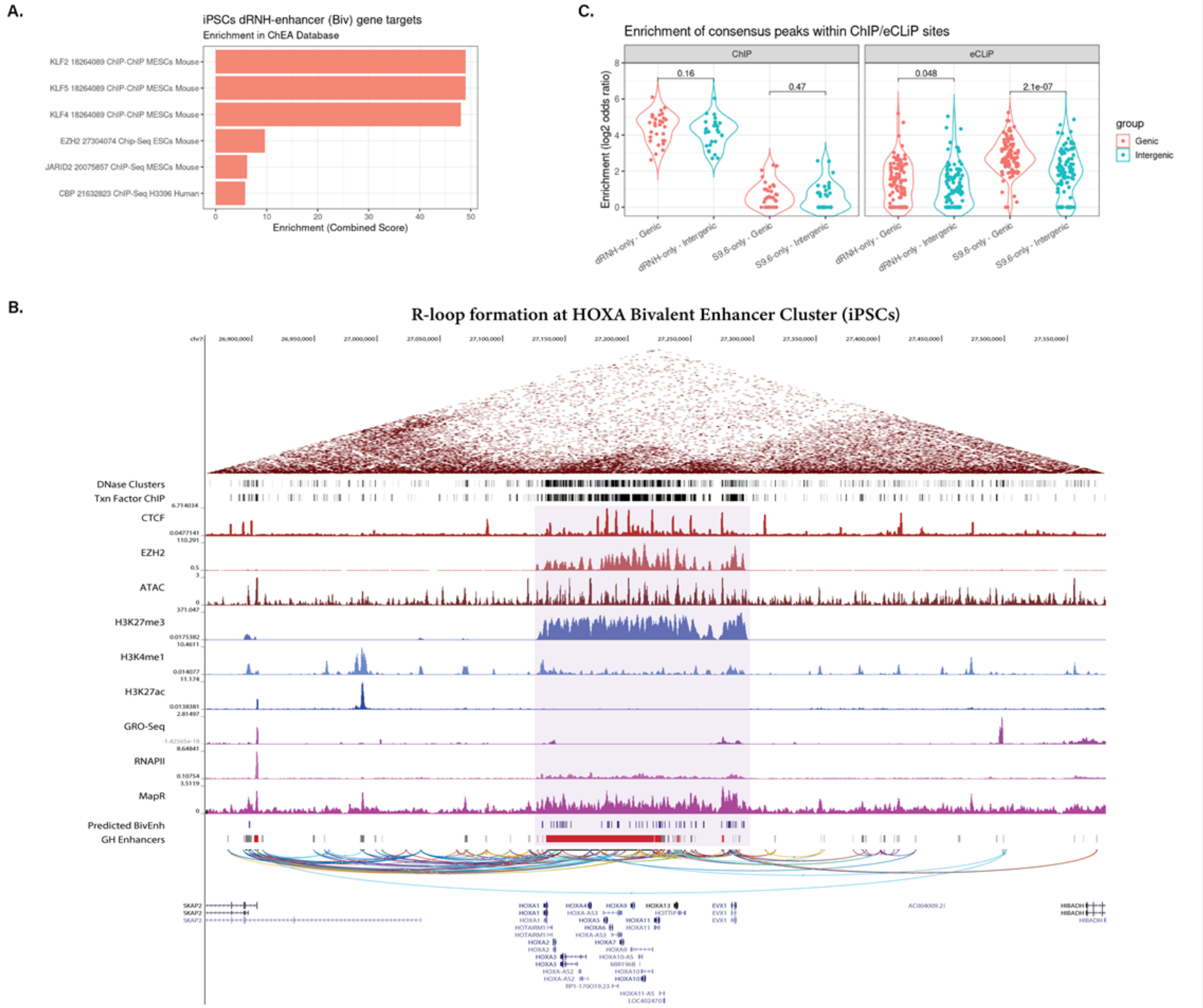

Figure S8. Analysis of bivalent enhancers in iPSCs and ChIP/eCLiP datasets. (A) Bar chart depicting the enrichment of gene targets of distal, bivalent enhancers with MapR peaks. Enrichment uses the ChEA 2016 database. (B) Representative genome browser image displaying the HOXA bivalent enhancer cluster. The genome browser session for this visualization is also provided within this manuscript (see **Availability**). (C) Distribution plot depicting the enrichment of consensus R-loop peaks within RNA binding protein ChIP-Seq and eCLiP-Seq peaks. R-loop consensus peaks are split into genic and intergenic groups. P values generated via Wilcoxon rank sum test.

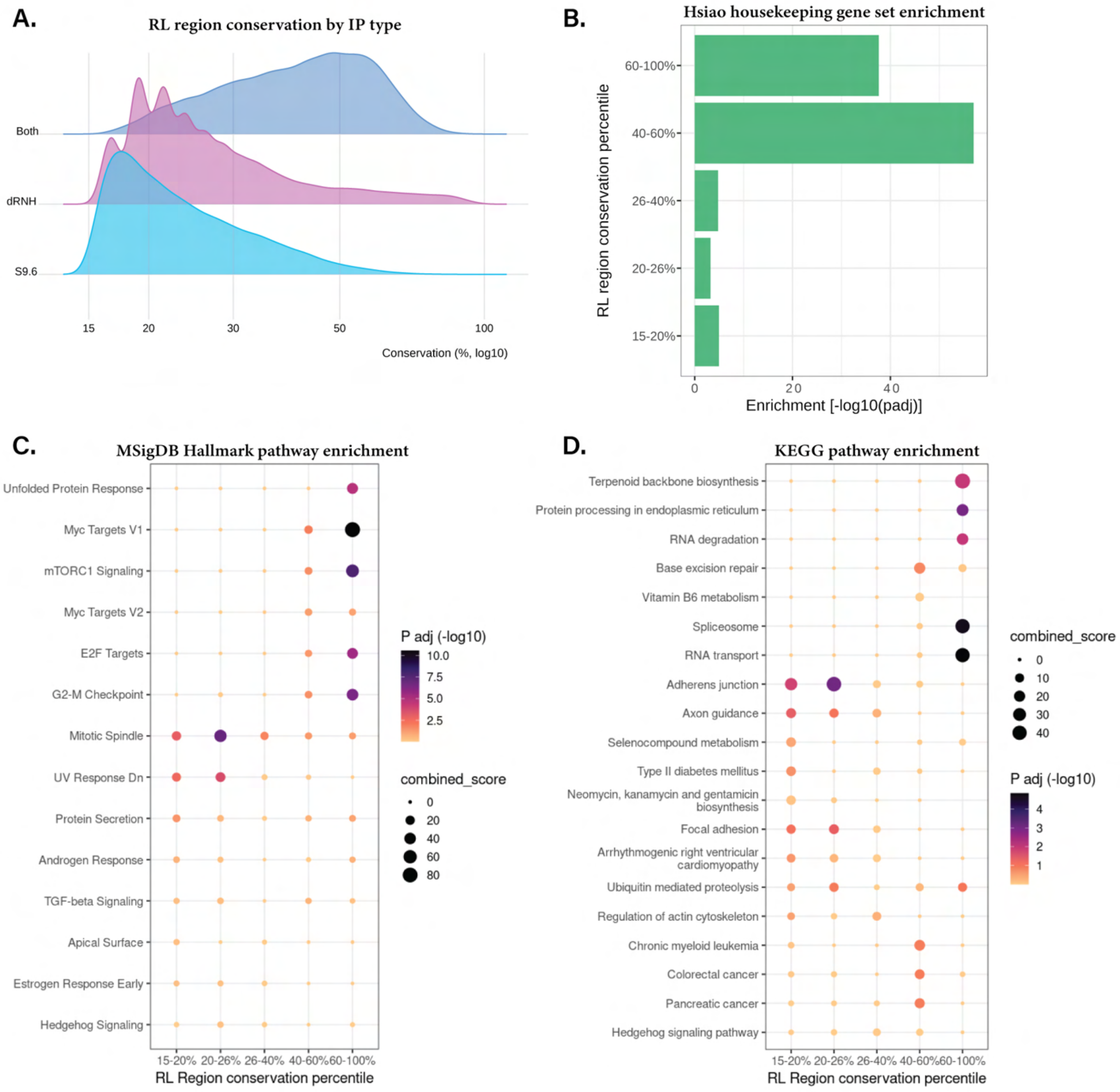

Figure S9. Extension of Figure 9. (A) Ridge plot showing the distribution of conservation percentages among the RL regions discovered by dRNH samples, S9.6 samples, or both. (B) Bar plot showing the enrichment of genes overlapping RL regions from each conservation bin within the “HSIAO_HOUSEKEEPING_GENES” gene set (MSigDB C2 collection). P value is from the hypergeometric test with Benjamini Hochberg correction for multiple testing. (C) Pathway enrichment plot showing the significance (via P adjusted value) and effect size (via Combined Score) of MSigDB Hallmark pathway enrichment from the genes overlapping RL regions within each percentile. (D) Same as (C) except with KEGG pathways.
